# Supplementary figures and images for: Digestibility of dinosaur food plants revisited and expanded: Previous data, new taxa, microbe donors, foliage maturity, and seasonality
Source: PLoS One. 2023 Dec 15;18(12):e0291058. doi: 10.1371/journal.pone.0291058 (PMC10723699; doi:10.1371/journal.pone.0291058)

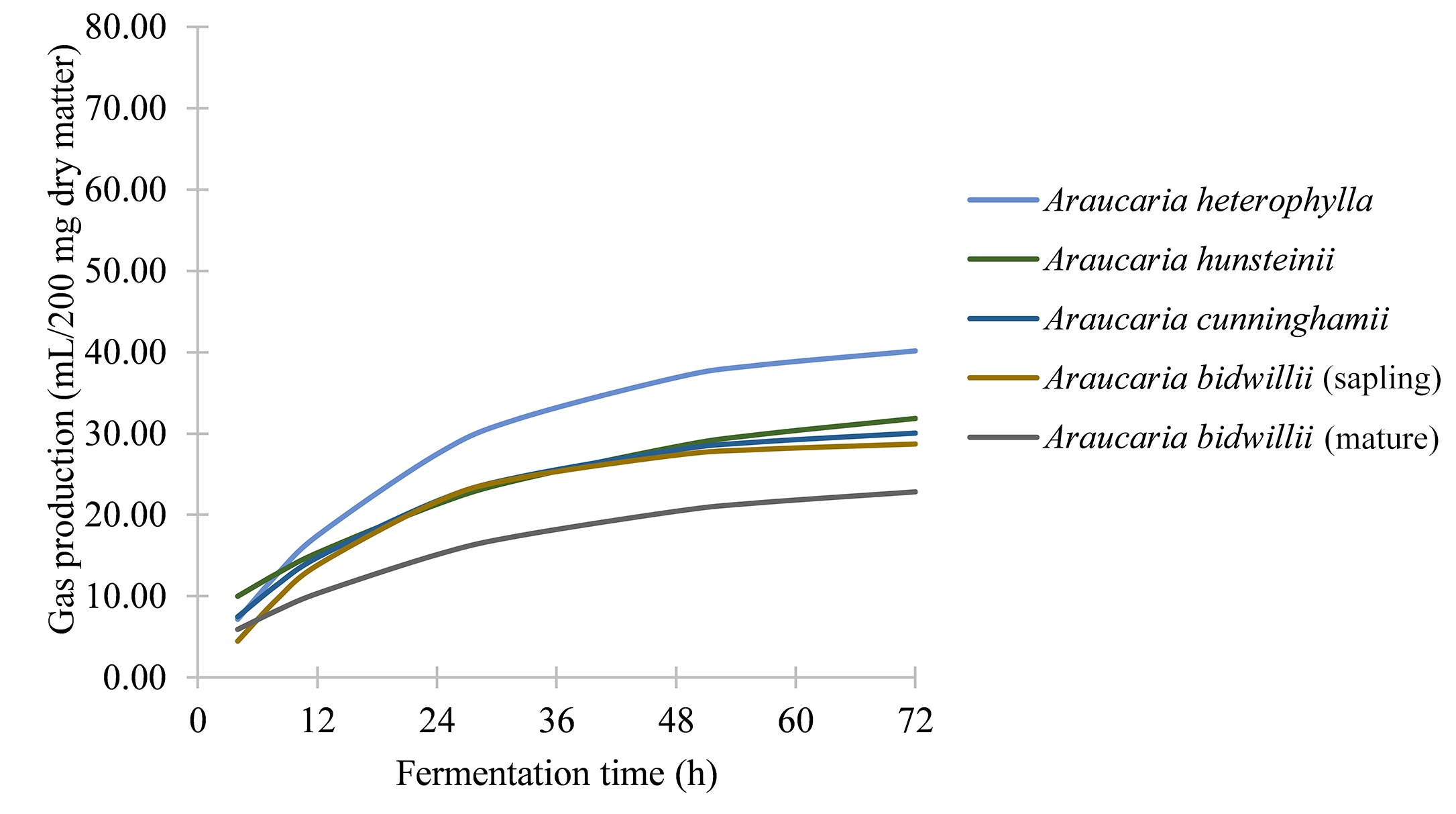

Supplement: S1 Fig — Araucariaceae spp. were collected in Australia in 2007 (Gee, unpubl. data). (TIF) [file pone.0291058.s001.tif]
