# Supplementary material for: Digestibility of dinosaur food plants revisited and expanded: Previous data, new taxa, microbe donors, foliage maturity, and seasonality
Source: PLoS One. 2023 Dec 15;18(12):e0291058. doi: 10.1371/journal.pone.0291058 (PMC10723699; doi:10.1371/journal.pone.0291058)
Supplement: S1 Table — Araucariaceae spp. were collected from naturally growing forest trees by CTG from Northern Queensland, Australia, in 2007. For each species, cumulative gas production up to 72 h and fermentative parameters in which a + b is maximum gas production (ml/200 mg DM), and c is rate of gas production are given (Gee, unpubl. data). (DOCX) [file pone.0291058.s002.docx]

|  | Cumulative gas production (ml/200 mg dry matter) | | | | | | | |  | |  |  |
| --- | --- | --- | --- | --- | --- | --- | --- | --- | --- | --- | --- | --- |
| Species | 4 h | 8 h | 12 h | 24 h | 32 h | 48 h | 56 h | 72 h | *a + b* | *c* | | |
| *Araucaria bidwillii* (mature) | 5.9 | 8.3 | 10.3 | 15.1 | 17.4 | 20.4 | 21.4 | 22.8 | 24.8 | 0.033 | | |
| *Araucaria bidwillii* (sapling) | 4.5 | 9.7 | 13.8 | 21.6 | 24.5 | 27.3 | 28.0 | 28.7 | 29.1 | 0.060 | | |
| *Araucaria cunninghamii* | 7.5 | 11.5 | 14.8 | 21.7 | 24.6 | 28.0 | 28.9 | 30.1 | 31.1 | 0.046 | | |
| *Araucaria hunsteinii* | 10.0 | 12.9 | 15.4 | 21.3 | 24.3 | 28.4 | 29.8 | 31.9 | 35.2 | 0.030 | | |
| *Araucaria heterophylla* | 7.2 | 12.8 | 17.5 | 27.5 | 31.8 | 36.9 | 38.4 | 40.2 | 41.9 | 0.044 | | |
