# Supplementary material for: Digestibility of dinosaur food plants revisited and expanded: Previous data, new taxa, microbe donors, foliage maturity, and seasonality
Source: PLoS One. 2023 Dec 15;18(12):e0291058. doi: 10.1371/journal.pone.0291058 (PMC10723699; doi:10.1371/journal.pone.0291058)
Supplement: S2 Table — A. Means and standard deviations (SD) for all samples as a seasonal average (Combined), and for the individual fall and spring datasets. B. Results of Tukey post hoc tests for species-species comparisons. C. ANOVA results for variables influencing gas production, including species, genus, family, rumen fluid donor, season, and maturity. D. Post hoc Tukey test results for the significance of season on gas production. The variable is the sample in the spring compared to the same sample in the fall. E. Results of Tukey post hoc test for comparisons of genera within the Araucariaceae family. F. Tukey post hoc results comparing gas production between families. G. Results of Tukey post hoc tests comparing significance of maturity in the species Cyathea cooperi and Marattia attenuata. H. Results of Tukey post hoc test for the interaction between season and maturity in the species Cyathea cooperi and Marattia attenuata. (ZIP) [file pone.0291058.s003.zip › S2 Table A.docx]

|  | Combined | |  | Spring | |  | Fall | |
| --- | --- | --- | --- | --- | --- | --- | --- | --- |
| Species | Mean | SD |  | Mean | SD |  | Mean | SD |
| *Angiopteris evecta* | 31.54 | 3.731482 |  | 29.81 | 2.745093025 |  | 33.28 | 3.930739661 |
| *Equisetum giganteum* | 53.26 | 8.601817 |  | 42.38 | 0.997914492 |  | 45.35 | 1.686218649 |
| *Osmunda regalis* | 22.18 | 6.762371 |  | 30.75 | 1.626345597 |  | 17.90 | 1.395229969 |
| *Marattia attenuata* (average) | 9.52 | 7.071957 |  | 10.41 | 5.558207145 |  | 8.85 | 8.343346365 |
| *Marattia attenuata* (young) | 14.77 | 1.241639 |  | 17.55 | 0.777817459 |  | 16.60 | 1.402378931 |
| *Marattia attenuata* (old) | 3.98 | 3.121698 |  | 6.85 | 0.635085296 |  | 1.10 | 0.541602560 |
| *Equisetum hyemale* | 58.33 | 3.453673 |  | 61.18 | 1.687947472 |  | 55.48 | 1.822772613 |
| *Cyathea cooperi* (average) | 21.47 | 2.373105 |  | 21.11 | 3.248928395 |  | 21.82 | 1.105505702 |
| *Cyathea cooperi* (old) | 17.38 | 0.550000 |  | 24.03 | 0.556027577 |  | 21.15 | 0.435889894 |
| *Cyathea cooperi* (young) | 20.35 | 1.604848 |  | 18.20 | 1.303840481 |  | 22.50 | 1.202774570 |
| *Araucaria laubenfelsii* | 22.75 | 1.393736 |  | 24.68 | 1.809926334 |  | 20.83 | 0.899536918 |
| *Araucaria columnaris* | 36.82 | 2.446572 |  | 28.45 | 1.126942767 |  | 25.30 | 0.503322296 |
| *Araucaria heterophylla "glauca"* | 29.90 | 1.210667 |  | 30.75 | 0.793725393 |  | 29.05 | 0.929157324 |
| *Araucaria bidwillii* | 21.60 | 5.383042 |  | 26.50 | 1.256980509 |  | 16.70 | 1.416568624 |
| *Agathis robusta* | 14.79 | 2.859789 |  | 17.43 | 0.607590871 |  | 12.15 | 0.404145188 |
| *Agathis lanceolata* | 24.14 | 4.366410 |  | 28.13 | 0.713559154 |  | 20.15 | 1.255653880 |
| *Agathis australis* | 18.94 | 1.156272 |  | 19.80 | 0.716472842 |  | 18.08 | 0.788986692 |
| *Wollemia nobilis* | 18.61 | 2.417954 |  | 19.86 | 2.803537306 |  | 17.36 | 1.047360628 |
