# Supplementary material for: Digestibility of dinosaur food plants revisited and expanded: Previous data, new taxa, microbe donors, foliage maturity, and seasonality
Source: PLoS One. 2023 Dec 15;18(12):e0291058. doi: 10.1371/journal.pone.0291058 (PMC10723699; doi:10.1371/journal.pone.0291058)
Supplement: S2 Table — A. Means and standard deviations (SD) for all samples as a seasonal average (Combined), and for the individual fall and spring datasets. B. Results of Tukey post hoc tests for species-species comparisons. C. ANOVA results for variables influencing gas production, including species, genus, family, rumen fluid donor, season, and maturity. D. Post hoc Tukey test results for the significance of season on gas production. The variable is the sample in the spring compared to the same sample in the fall. E. Results of Tukey post hoc test for comparisons of genera within the Araucariaceae family. F. Tukey post hoc results comparing gas production between families. G. Results of Tukey post hoc tests comparing significance of maturity in the species Cyathea cooperi and Marattia attenuata. H. Results of Tukey post hoc test for the interaction between season and maturity in the species Cyathea cooperi and Marattia attenuata. (ZIP) [file pone.0291058.s003.zip › S2 Table B.docx]

| Species Interaction | Difference | Lower Interval | Upper Interval | | *P*-value | |
| --- | --- | --- | --- | --- | --- | --- |
| *Marattia attenuata*-*Zostera marina* | 9.546429 | 2.182953 | 16.9099 | 0.001276 | |  |
| *Agathis robusta-Zostera marina* | 14.8125 | 6.859037 | 22.76596 | 1E-07 | |  |
| *Wollemia nobilis-Zostera marina* | 18.6375 | 11.37702 | 25.89799 | 0 | |  |
| *Agathis australis-Zostera marina* | 18.9625 | 11.00904 | 26.91596 | 0 | |  |
| *Cyathea cooperi-Zostera marina* | 21.49375 | 14.23327 | 28.75424 | 0 | |  |
| *Araucaria bidwillii-Zostera marina* | 21.625 | 13.67154 | 29.57846 | 0 | |  |
| *Osmunda regalis-Zostera marina* | 22.20833 | 13.82465 | 30.59202 | 0 | |  |
| *Araucaria laubenfelsii-Zostera marina* | 22.775 | 14.82154 | 30.72846 | 0 | |  |
| *Agathis lanceolata-Zostera marina* | 24.1625 | 16.20904 | 32.11596 | 0 | |  |
| *Araucaria columnaris-Zostera marina* | 26.9 | 18.94654 | 34.85346 | 0 | |  |
| *Araucaria heterophylla* "*glauca*"*-Zostera marina* | 29.925 | 21.97154 | 37.87846 | 0 | |  |
| *Angiopteris evecta-Zostera marina* | 31.56875 | 24.30827 | 38.82924 | 0 | |  |
| *Equisetum giganteum-Zostera marina* | 43.8875 | 35.93404 | 51.84096 | 0 | |  |
| Hay standard*-Zostera marina* | 44.345 | 35.63242 | 53.05758 | 0 | |  |
| Concentrate standard*-Zostera marina* | 56.425 | 47.71242 | 65.13758 | 0 | |  |
| *Equisetum hyemale-Zostera marina* | 58.35 | 50.39654 | 66.30346 | 0 | |  |
| *Agathis robusta-Marattia attenuata* | 5.266071 | -0.49022 | 11.02237 | 0.115348 | |  |
| *Wollemia nobilis-Marattia attenuata* | 9.091071 | 4.337968 | 13.84417 | 1E-07 | |  |
| *Agathis australis-Marattia attenuata* | 9.416071 | 3.659778 | 15.17237 | 6.8E-06 | |  |
| *Cyathea cooperi-Marattia attenuata* | 11.94732 | 7.194218 | 16.70042 | 0 | |  |
| *Araucaria bidwillii-Marattia attenuata* | 12.07857 | 6.322278 | 17.83487 | 0 | |  |
| *Osmunda regalis-Marattia attenuata* | 12.6619 | 6.324434 | 18.99938 | 0 | |  |
| *Araucaria laubenfelsii-Marattia attenuata* | 13.22857 | 7.472278 | 18.98487 | 0 | |  |
| *Agathis lanceolata-Marattia attenuata* | 14.61607 | 8.859778 | 20.37237 | 0 | |  |
| *Araucaria columnaris-Marattia attenuata* | 17.35357 | 11.59728 | 23.10987 | 0 | |  |
| *Araucaria heterophylla* "*glauca*"*-Marattia attenuata* | 20.37857 | 14.62228 | 26.13487 | 0 | |  |
| *Angiopteris evecta-Marattia attenuata* | 22.02232 | 17.26922 | 26.77542 | 0 | |  |
| *Equisetum giganteum-Marattia attenuata* | 34.34107 | 28.58478 | 40.09737 | 0 | |  |
| Hay standard*-Marattia attenuata* | 34.79857 | 28.032 | 41.56514 | 0 | |  |
| Concentrate standard*-Marattia attenuata* | 46.87857 | 40.112 | 53.64514 | 0 | |  |
| *Equisetum hyemale-Marattia attenuata* | 48.80357 | 43.04728 | 54.55987 | 0 | |  |
| *Wollemia nobilis-Agathis robusta* | 3.825 | -1.79895 | 9.448947 | 0.582872 | |  |
| *Agathis australis-Agathis robusta* | 4.15 | -2.34398 | 10.64398 | 0.687917 | |  |
| *Cyathea cooperi-Agathis robusta* | 6.68125 | 1.057303 | 12.3052 | 0.005499 | |  |
| *Araucaria bidwillii-Agathis robusta* | 6.8125 | 0.318525 | 13.30648 | 0.029395 | |  |
| *Osmunda regalis-Agathis robusta* | 7.395833 | 0.381539 | 14.41013 | 0.027685 | |  |
| *Araucaria laubenfelsii-Agathis robusta* | 7.9625 | 1.468525 | 14.45648 | 0.003336 | |  |
| *Agathis lanceolata-Agathis robusta* | 9.35 | 2.856025 | 15.84398 | 0.000156 | |  |
| *Araucaria columnaris-Agathis robusta* | 12.0875 | 5.593525 | 18.58148 | 1E-07 | |  |
| *Araucaria heterophylla* "*glauca*"*-Agathis robusta* | 15.1125 | 8.618525 | 21.60648 | 0 | |  |
| *Angiopteris evecta-Agathis robusta* | 16.75625 | 11.1323 | 22.3802 | 0 | |  |
| *Equisetum giganteum-Agathis robusta* | 29.075 | 22.58102 | 35.56898 | 0 | |  |
| Hay standard*-Agathis robusta* | 29.5325 | 22.12823 | 36.93677 | 0 | |  |
| Concentrate standard*-Agathis robusta* | 41.6125 | 34.20823 | 49.01677 | 0 | |  |
| *Equisetum hyemale-Agathis robusta* | 43.5375 | 37.04352 | 50.03148 | 0 | |  |
| *Agathis australis-Wollemia nobilis* | 0.325 | -5.29895 | 5.948947 | 1 | |  |
| *Cyathea cooperi-Wollemia nobilis* | 2.85625 | -1.73568 | 7.448184 | 0.729098 | |  |
| *Araucaria bidwillii-Wollemia nobilis* | 2.9875 | -2.63645 | 8.611447 | 0.901685 | |  |
| *Osmunda regalis-Wollemia nobilis* | 3.570833 | -2.64667 | 9.788341 | 0.831172 | |  |
| *Araucaria laubenfelsii-Wollemia nobilis* | 4.1375 | -1.48645 | 9.761447 | 0.439533 | |  |
| *Agathis lanceolata-Wollemia nobilis* | 5.525 | -0.09895 | 11.14895 | 0.05998 | |  |
| *Araucaria columnaris-Wollemia nobilis* | 8.2625 | 2.638553 | 13.88645 | 9.92E-05 | |  |
| *Araucaria heterophylla* "*glauca*"*-Wollemia nobilis* | 11.2875 | 5.663553 | 16.91145 | 0 | |  |
| *Angiopteris evecta-Wollemia nobilis* | 12.93125 | 8.339316 | 17.52318 | 0 | |  |
| *Equisetum giganteum-Wollemia nobilis* | 25.25 | 19.62605 | 30.87395 | 0 | |  |
| Hay standard*-Wollemia nobilis* | 25.7075 | 19.05316 | 32.36184 | 0 | |  |
| Concentrate standard*-Wollemia nobilis* | 37.7875 | 31.13316 | 44.44184 | 0 | |  |
| *Equisetum hyemale-Wollemia nobilis* | 39.7125 | 34.08855 | 45.33645 | 0 | |  |
| *Cyathea cooperi-Agathis australis* | 2.53125 | -3.0927 | 8.155197 | 0.975487 | |  |
| *Araucaria bidwillii-Agathis australis* | 2.6625 | -3.83148 | 9.156475 | 0.990154 | |  |
| *Osmunda regalis-Agathis australis* | 3.245833 | -3.76846 | 10.26013 | 0.968398 | |  |
| *Araucaria laubenfelsii-Agathis australis* | 3.8125 | -2.68148 | 10.30648 | 0.8061 | |  |
| *Agathis lanceolata-Agathis australis* | 5.2 | -1.29398 | 11.69398 | 0.2907 | |  |
| *Araucaria columnaris-Agathis australis* | 7.9375 | 1.443525 | 14.43148 | 0.003511 | |  |
| *Araucaria heterophylla* "*glauca*"*-Agathis australis* | 10.9625 | 4.468525 | 17.45648 | 2.8E-06 | |  |
| *Angiopteris evecta-Agathis australis* | 12.60625 | 6.982303 | 18.2302 | 0 | |  |
| *Equisetum giganteum-Agathis australis* | 24.925 | 18.43102 | 31.41898 | 0 | |  |
| Hay standard*-Agathis australis* | 25.3825 | 17.97823 | 32.78677 | 0 | |  |
| Concentrate standard*-Agathis australis* | 37.4625 | 30.05823 | 44.86677 | 0 | |  |
| *Equisetum hyemale-Agathis australis* | 39.3875 | 32.89352 | 45.88148 | 0 | |  |
| *Araucaria bidwillii-Cyathea cooperi* | 0.13125 | -5.4927 | 5.755197 | 1 | |  |
| *Osmunda regalis-Cyathea cooperi* | 0.714583 | -5.50292 | 6.932091 | 1 | |  |
| *Araucaria laubenfelsii-Cyathea cooperi* | 1.28125 | -4.3427 | 6.905197 | 0.999993 | |  |
| *Agathis lanceolata-Cyathea cooperi* | 2.66875 | -2.9552 | 8.292697 | 0.9605 | |  |
| *Araucaria columnaris-Cyathea cooperi* | 5.40625 | -0.2177 | 11.0302 | 0.074158 | |  |
| *Araucaria heterophylla* "*glauca*"*-Cyathea cooperi* | 8.43125 | 2.807303 | 14.0552 | 6.21E-05 | |  |
| *Angiopteris evecta-Cyathea cooperi* | 10.075 | 5.483066 | 14.66693 | 0 | |  |
| *Equisetum giganteum-Cyathea cooperi* | 22.39375 | 16.7698 | 28.0177 | 0 | |  |
| Hay standard-*Cyathea cooperi* | 22.85125 | 16.19691 | 29.50559 | 0 | |  |
| Concentrate standard*-Cyathea cooperi* | 34.93125 | 28.27691 | 41.58559 | 0 | |  |
| *Equisetum hyemale-Cyathea cooperi* | 36.85625 | 31.2323 | 42.4802 | 0 | |  |
| *Osmunda regalis-Araucaria bidwillii* | 0.583333 | -6.43096 | 7.597628 | 1 | |  |
| *Araucaria laubenfelsii-Araucaria bidwillii* | 1.15 | -5.34398 | 7.643975 | 1 | |  |
| *Agathis lanceolata-Araucaria bidwillii* | 2.5375 | -3.95648 | 9.031475 | 0.994045 | |  |
| *Araucaria columnaris-Araucaria bidwillii* | 5.275 | -1.21898 | 11.76898 | 0.267707 | |  |
| *Araucaria heterophylla* "*glauca*"*-Araucaria bidwillii* | 8.3 | 1.806025 | 14.79398 | 0.001647 | |  |
| *Angiopteris evecta-Araucaria bidwillii* | 9.94375 | 4.319803 | 15.5677 | 7E-07 | |  |
| *Equisetum giganteum-Araucaria bidwillii* | 22.2625 | 15.76852 | 28.75648 | 0 | |  |
| Hay standard-*Araucaria bidwillii* | 22.72 | 15.31573 | 30.12427 | 0 | |  |
| Concentrate standard*-Araucaria bidwillii* | 34.8 | 27.39573 | 42.20427 | 0 | |  |
| *Equisetum hyemale-Araucaria bidwillii* | 36.725 | 30.23102 | 43.21898 | 0 | |  |
| *Araucaria laubenfelsii-Osmunda regalis* | 0.566667 | -6.44763 | 7.580961 | 1 | |  |
| *Agathis lanceolata-Osmunda regalis* | 1.954167 | -5.06013 | 8.968461 | 0.999896 | |  |
| *Araucaria columnaris-Osmunda regalis* | 4.691667 | -2.32263 | 11.70596 | 0.612164 | |  |
| *Araucaria heterophylla* "*glauca*"*-Osmunda regalis* | 7.716667 | 0.702372 | 14.73096 | 0.016322 | |  |
| *Angiopteris evecta-Osmunda regalis* | 9.360417 | 3.142909 | 15.57792 | 5.62E-05 | |  |
| *Equisetum giganteum-Osmunda regalis* | 21.67917 | 14.66487 | 28.69346 | 0 | |  |
| Hay standard*-Osmunda regalis* | 22.13667 | 14.27207 | 30.00126 | 0 | |  |
| Concentrate standard*-Osmunda regalis* | 34.21667 | 26.35207 | 42.08126 | 0 | |  |
| *Equisetum hyemale-Osmunda regalis* | 36.14167 | 29.12737 | 43.15596 | 0 | |  |
| *Agathis lanceolata-Araucaria laubenfelsii* | 1.3875 | -5.10648 | 7.881475 | 0.999997 | |  |
| *Araucaria columnaris-Araucaria laubenfelsii* | 4.125 | -2.36898 | 10.61898 | 0.697388 | |  |
| *Araucaria heterophylla* "*glauca*"*-Araucaria laubenfelsii* | 7.15 | 0.656025 | 13.64398 | 0.016151 | |  |
| *Angiopteris evecta-Araucaria laubenfelsii* | 8.79375 | 3.169803 | 14.4177 | 2.22E-05 | |  |
| *Equisetum giganteum-Araucaria laubenfelsii* | 21.1125 | 14.61852 | 27.60648 | 0 | |  |
| Hay standard*-Araucaria laubenfelsii* | 21.57 | 14.16573 | 28.97427 | 0 | |  |
| Concentrate standard*-Araucaria laubenfelsii* | 33.65 | 26.24573 | 41.05427 | 0 | |  |
| *Equisetum hyemale-Araucaria laubenfelsii* | 35.575 | 29.08102 | 42.06898 | 0 | |  |
| *Araucaria columnaris-Agathis lanceolata* | 2.7375 | -3.75648 | 9.231475 | 0.986964 | |  |
| *Araucaria heterophylla* "*glauca*"*-Agathis lanceolata* | 5.7625 | -0.73148 | 12.25648 | 0.147261 | |  |
| *Angiopteris evecta-Agathis lanceolata* | 7.40625 | 1.782303 | 13.0302 | 0.000956 | |  |
| *Equisetum giganteum-Agathis lanceolata* | 19.725 | 13.23102 | 26.21898 | 0 | |  |
| Hay standard*-Agathis lanceolata* | 20.1825 | 12.77823 | 27.58677 | 0 | |  |
| Concentrate standard*-Agathis lanceolata* | 32.2625 | 24.85823 | 39.66677 | 0 | |  |
| *Equisetum hyemale-Agathis lanceolata* | 34.1875 | 27.69352 | 40.68148 | 0 | |  |
| *Araucaria heterophylla* "*glauca*"*-Araucaria columnaris* | 3.025 | -3.46898 | 9.518975 | 0.966466 | |  |
| *Angiopteris evecta-Araucaria columnaris* | 4.66875 | -0.9552 | 10.2927 | 0.234462 | |  |
| *Equisetum giganteum-Araucaria columnaris* | 16.9875 | 10.49352 | 23.48148 | 0 | |  |
| Hay standard*-Araucaria columnaris* | 17.445 | 10.04073 | 24.84927 | 0 | |  |
| Concentrate standard*-Araucaria columnaris* | 29.525 | 22.12073 | 36.92927 | 0 | |  |
| *Equisetum hyemale-Araucaria columnaris* | 31.45 | 24.95602 | 37.94398 | 0 | |  |
| *Angiopteris evecta-Araucaria heterophylla* "*glauca*" | 1.64375 | -3.9802 | 7.267697 | 0.999807 | |  |
| *Equisetum giganteum-Araucaria heterophylla* "*glauca*" | 13.9625 | 7.468525 | 20.45648 | 0 | |  |
| Hay standard*-Araucaria heterophylla* "*glauca*" | 14.42 | 7.015729 | 21.82427 | 0 | |  |
| Concentrate standard*-Araucaria heterophylla* "*glauca*" | 26.5 | 19.09573 | 33.90427 | 0 | |  |
| *Equisetum hyemale-Araucaria heterophylla* "*glauca*" | 28.425 | 21.93102 | 34.91898 | 0 | |  |
| *Equisetum giganteum-Angiopteris evecta* | 12.31875 | 6.694803 | 17.9427 | 0 | |  |
| Hay standard*-Angiopteris evecta* | 12.77625 | 6.121906 | 19.43059 | 0 | |  |
| Concentrate standard*-Angiopteris evecta* | 24.85625 | 18.20191 | 31.51059 | 0 | |  |
| *Equisetum hyemale-Angiopteris evecta* | 26.78125 | 21.1573 | 32.4052 | 0 | |  |
| Hay standard*-Equisetum giganteum* | 0.4575 | -6.94677 | 7.861771 | 1 | |  |
| Concentrate standard*-Equisetum giganteum* | 12.5375 | 5.133229 | 19.94177 | 2.6E-06 | |  |
| *Equisetum hyemale-Equisetum giganteum* | 14.4625 | 7.968525 | 20.95648 | 0 | |  |
| Concentrate standard*-*Hay standard | 12.08 | 3.865699 | 20.2943 | 0.000097 | |  |
| *Equisetum hyemale-*Hay standard | 14.005 | 6.600729 | 21.40927 | 1E-07 | |  |
| *Equisetum hyemale-*Concentrate standard | 1.925 | -5.47927 | 9.329271 | 0.999958 | |  |
