# Supplementary material for: Digestibility of dinosaur food plants revisited and expanded: Previous data, new taxa, microbe donors, foliage maturity, and seasonality
Source: PLoS One. 2023 Dec 15;18(12):e0291058. doi: 10.1371/journal.pone.0291058 (PMC10723699; doi:10.1371/journal.pone.0291058)
Supplement: S3 Table — Parameters given are A, B, and c, with the estimate, standard error (SE), alpha, lower interval, upper interval, t-value, probability for each parameter of each sample. (DOCX) [file pone.0291058.s004.docx]

| **Parameter** | **Estimate** | **SE** | **Alpha** | **Lower Int.** | **Upper Int.** | ***t*-Value** | **Probability** | **Sample** |
| --- | --- | --- | --- | --- | --- | --- | --- | --- |
| A | 2.52 | 0.1877 | 0.05 | 2.1331 | 2.8968 | 13.4 | <.0001 | Spring *Zostera marina* |
| B | 60.70 | 77.5745 | 0.05 | -97.1311 | 218.5 | 0.78 | 0.4395 | Spring *Zostera marina* |
| c | 0.00 | 0.00222 | 0.05 | -0.00291 | 0.00613 | 0.72 | 0.4738 | Spring *Zostera marina* |
| A | -0.06 | 1.9475 | 0.05 | -4.0266 | 3.8976 | -0.03 | 0.9738 | Spring *Angiopteris evecta* (1) |
| B | 42.06 | 1.8493 | 0.05 | 38.299 | 45.8237 | 22.74 | <.0001 | Spring *Angiopteris evecta* (1) |
| c | 0.10 | 0.0071 | 0.05 | 0.0809 | 0.1098 | 13.43 | <.0001 | Spring *Angiopteris evecta* (1) |
| A | -1.48 | 1.8629 | 0.05 | -5.2715 | 2.3086 | -0.8 | 0.4322 | Spring *Angiopteris evecta* (2) |
| B | 38.81 | 1.7749 | 0.05 | 35.1971 | 42.4192 | 21.87 | <.0001 | Spring *Angiopteris evecta* (2) |
| c | 0.10 | 0.00746 | 0.05 | 0.084 | 0.1144 | 13.29 | <.0001 | Spring *Angiopteris evecta* (2) |
| A | -8.38 | 1.4969 | 0.05 | -11.4216 | -5.3305 | -5.6 | <.0001 | Spring *Equisetum giganteum* |
| B | 61.54 | 1.3959 | 0.05 | 58.7012 | 64.3809 | 44.09 | <.0001 | Spring *Equisetum giganteum* |
| c | 0.08 | 0.00348 | 0.05 | 0.0716 | 0.0858 | 22.63 | <.0001 | Spring *Equisetum giganteum* |
| A | 2.75 | 1.0226 | 0.05 | 0.5696 | 4.9288 | 2.69 | 0.0168 | Spring *Osmunda regalis* |
| B | 39.11 | 0.9207 | 0.05 | 37.1496 | 41.0744 | 42.48 | <.0001 | Spring *Osmunda regalis* |
| c | 0.05 | 0.00315 | 0.05 | 0.0391 | 0.0526 | 14.55 | <.0001 | Spring *Osmunda regalis* |
| A | 4.66 | 0.4845 | 0.05 | 3.6272 | 5.6927 | 9.62 | <.0001 | Spring *Marattia attenuata* (young) |
| B | 25.84 | 0.6139 | 0.05 | 24.5315 | 27.1487 | 42.09 | <.0001 | Spring *Marattia attenuata* (young) |
| c | 0.03 | 0.00215 | 0.05 | 0.0232 | 0.0324 | 12.96 | <.0001 | Spring *Marattia attenuata* (young) |
| A | 3.61 | 0.311 | 0.05 | 2.9755 | 4.2408 | 11.6 | <.0001 | Spring *Marattia attenuata* (old) |
| B | 17.95 | 0.9779 | 0.05 | 15.9646 | 19.9436 | 18.36 | <.0001 | Spring *Marattia attenuata* (old) |
| c | 0.02 | 0.00218 | 0.05 | 0.0128 | 0.0217 | 7.9 | <.0001 | Spring *Marattia attenuata* (old) |
| A | -9.64 | 1.8552 | 0.05 | -13.4147 | -5.8658 | -5.2 | <.0001 | Spring *Equisetum hyemale* |
| B | 81.75 | 1.7176 | 0.05 | 78.2529 | 85.2417 | 47.6 | <.0001 | Spring *Equisetum hyemale* |
| c | 0.07 | 0.00317 | 0.05 | 0.0671 | 0.08 | 23.22 | <.0001 | Spring *Equisetum hyemale* |
| A | 2.48 | 0.7383 | 0.05 | 0.9759 | 3.9801 | 3.36 | 0.002 | Spring *Cyathea cooperi* (old) |
| B | 33.43 | 0.6773 | 0.05 | 32.0477 | 34.8038 | 49.35 | <.0001 | Spring *Cyathea cooperi* (old) |
| c | 0.04 | 0.0026 | 0.05 | 0.0358 | 0.0464 | 15.8 | <.0001 | Spring *Cyathea cooperi* (old) |
| A | 6.46 | 0.7345 | 0.05 | 4.961 | 7.9497 | 8.79 | <.0001 | Spring *Cyathea cooperi* (young) |
| B | 23.30 | 0.6727 | 0.05 | 21.9274 | 24.6648 | 34.63 | <.0001 | Spring *Cyathea cooperi* (young) |
| c | 0.04 | 0.00372 | 0.05 | 0.0338 | 0.0489 | 11.13 | <.0001 | Spring *Cyathea cooperi* (young) |
| A | -1.45 | 1.2597 | 0.05 | -4.0159 | 1.11 | -1.15 | 0.257 | Spring *Araucaria laubenfelsii* |
| B | 37.12 | 1.1341 | 0.05 | 34.8162 | 39.431 | 32.73 | <.0001 | Spring *Araucaria laubenfelsii* |
| c | 0.05 | 0.00429 | 0.05 | 0.0462 | 0.0636 | 12.78 | <.0001 | Spring *Araucaria laubenfelsii* |
| A | 1.91 | 1.4031 | 0.05 | -0.9466 | 4.7628 | 1.36 | 0.1831 | Spring *Araucaria columnaris* |
| B | 36.99 | 1.2628 | 0.05 | 34.4159 | 39.5541 | 29.29 | <.0001 | Spring *Araucaria columnaris* |
| c | 0.05 | 0.00479 | 0.05 | 0.0448 | 0.0643 | 11.38 | <.0001 | Spring *Araucaria columnaris* |
| A | 4.37 | 0.7658 | 0.05 | 2.8094 | 5.9255 | 5.7 | <.0001 | Spring *Araucaria heterophylla* |
| B | 37.96 | 0.6918 | 0.05 | 36.551 | 39.3659 | 54.87 | <.0001 | Spring *Araucaria heterophylla* |
| c | 0.04 | 0.00241 | 0.05 | 0.0395 | 0.0494 | 18.41 | <.0001 | Spring *Araucaria heterophylla* |
| A | 1.76 | 1.1273 | 0.05 | -0.537 | 4.05 | 1.56 | 0.1287 | Spring *Araucaria bidwillii* |
| B | 35.64 | 1.0252 | 0.05 | 33.5493 | 37.7207 | 34.76 | <.0001 | Spring *Araucaria bidwillii* |
| c | 0.06 | 0.00416 | 0.05 | 0.0535 | 0.0705 | 14.9 | <.0001 | Spring *Araucaria bidwillii* |
| A | 6.84 | 0.4093 | 0.05 | 6.0083 | 7.6739 | 16.71 | <.0001 | Spring *Agathis robusta* |
| B | 22.70 | 0.4981 | 0.05 | 21.6893 | 23.716 | 45.58 | <.0001 | Spring *Agathis robusta* |
| c | 0.03 | 0.00206 | 0.05 | 0.0244 | 0.0328 | 13.88 | <.0001 | Spring *Agathis robusta* |
| A | 7.92 | 0.6226 | 0.05 | 6.6565 | 9.1901 | 12.73 | <.0001 | Spring *Agathis lanceolata* |
| B | 31.59 | 0.5706 | 0.05 | 30.4321 | 32.754 | 55.37 | <.0001 | Spring *Agathis lanceolata* |
| c | 0.04 | 0.00232 | 0.05 | 0.0365 | 0.046 | 17.77 | <.0001 | Spring *Agathis lanceolata* |
| A | 5.02 | 0.7922 | 0.05 | 3.4044 | 6.6278 | 6.33 | <.0001 | Spring *Agathis australis* |
| B | 25.66 | 0.7108 | 0.05 | 24.2116 | 27.104 | 36.1 | <.0001 | Spring *Agathis australis* |
| c | 0.05 | 0.0038 | 0.05 | 0.0422 | 0.0577 | 13.14 | <.0001 | Spring *Agathis australis* |
| A | 3.57 | 0.7879 | 0.05 | 1.9679 | 5.1737 | 4.53 | <.0001 | Spring *Wollemia nobilis* (1) |
| B | 25.37 | 0.7212 | 0.05 | 23.9069 | 26.8414 | 35.19 | <.0001 | Spring *Wollemia nobilis* (1) |
| c | 0.04 | 0.00366 | 0.05 | 0.034 | 0.0489 | 11.32 | <.0001 | Spring *Wollemia nobilis* (1) |
| A | 2.55 | 0.8853 | 0.05 | 0.7479 | 4.3501 | 2.88 | 0.0069 | Spring *Wollemia nobilis* (2) |
| B | 31.76 | 0.8168 | 0.05 | 30.102 | 33.4258 | 38.89 | <.0001 | Spring *Wollemia nobilis* (2) |
| c | 0.04 | 0.00327 | 0.05 | 0.0337 | 0.047 | 12.32 | <.0001 | Spring *Wollemia nobilis* (2) |
| A | 0.26 | 2.7255 | 0.05 | -5.2852 | 5.8049 | 0.1 | 0.9246 | Fall *Angiopteris evecta* (1) |
| B | 38.49 | 2.6199 | 0.05 | 33.1558 | 43.8163 | 14.69 | <.0001 | Fall *Angiopteris evecta* (1) |
| c | 0.11 | 0.0115 | 0.05 | 0.0884 | 0.1352 | 9.73 | <.0001 | Fall *Angiopteris evecta* (1) |
| A | 3.45 | 2.5224 | 0.05 | -1.6867 | 8.5768 | 1.37 | 0.1812 | Fall *Angiopteris evecta* (2) |
| B | 42.40 | 2.3891 | 0.05 | 37.5402 | 47.2615 | 17.75 | <.0001 | Fall *Angiopteris evecta* (2) |
| c | 0.09 | 0.00902 | 0.05 | 0.0742 | 0.1109 | 10.26 | <.0001 | Fall *Angiopteris evecta* (2) |
| A | -8.11 | 1.8291 | 0.05 | -11.8297 | -4.3871 | -4.43 | <.0001 | Fall *Equisetum giganteum* |
| B | 63.52 | 1.7234 | 0.05 | 60.0169 | 67.0293 | 36.86 | <.0001 | Fall *Equisetum giganteum* |
| c | 0.09 | 0.00428 | 0.05 | 0.0787 | 0.0961 | 20.44 | <.0001 | Fall *Equisetum giganteum* |
| A | 4.01 | 0.6666 | 0.05 | 2.6525 | 5.3648 | 6.01 | <.0001 | Fall *Osmunda regalis* |
| B | 25.06 | 0.6034 | 0.05 | 23.8368 | 26.2919 | 41.54 | <.0001 | Fall *Osmunda regalis* |
| c | 0.04 | 0.00317 | 0.05 | 0.0374 | 0.0503 | 13.82 | <.0001 | Fall *Osmunda regalis* |
| A | 4.86 | 0.8908 | 0.05 | 3.0475 | 6.6723 | 5.46 | <.0001 | Fall *Marattia attenuata* (young) |
| B | 24.35 | 1.0657 | 0.05 | 22.1786 | 26.5151 | 22.85 | <.0001 | Fall *Marattia attenuata* (young) |
| c | 0.03 | 0.00418 | 0.05 | 0.0205 | 0.0375 | 6.93 | <.0001 | Fall *Marattia attenuata* (young) |
| A | 4.16 | 0.2319 | 0.05 | 3.692 | 4.6355 | 17.96 | <.0001 | Fall *Marattia attenuata* (old) |
| B | 10.10 | 0.7586 | 0.05 | 8.56 | 11.6469 | 13.32 | <.0001 | Fall *Marattia attenuata* (old) |
| c | 0.02 | 0.00291 | 0.05 | 0.011 | 0.0228 | 5.81 | <.0001 | Fall *Marattia attenuata* (old) |
| A | -7.29 | 2.0088 | 0.05 | -11.3803 | -3.2067 | -3.63 | 0.0009 | Fall *Equisetum hyemale* |
| B | 73.92 | 1.8372 | 0.05 | 70.18 | 77.6554 | 40.23 | <.0001 | Fall *Equisetum hyemale* |
| c | 0.07 | 0.00364 | 0.05 | 0.0582 | 0.073 | 18 | <.0001 | Fall *Equisetum hyemale* |
| A | 4.57 | 0.661 | 0.05 | 3.2202 | 5.9098 | 6.91 | <.0001 | Fall *Cyathea cooperi* (old) |
| B | 27.72 | 0.5939 | 0.05 | 26.5156 | 28.9322 | 46.68 | <.0001 | Fall *Cyathea cooperi* (old) |
| c | 0.05 | 0.00289 | 0.05 | 0.0413 | 0.0531 | 16.32 | <.0001 | Fall *Cyathea cooperi* (old) |
| A | 4.92 | 0.9398 | 0.05 | 3.012 | 6.836 | 5.24 | <.0001 | Fall *Cyathea cooperi* (young) |
| B | 28.55 | 0.8434 | 0.05 | 26.8311 | 30.2629 | 33.85 | <.0001 | Fall *Cyathea cooperi* (young) |
| c | 0.05 | 0.00403 | 0.05 | 0.0408 | 0.0572 | 12.14 | <.0001 | Fall *Cyathea cooperi* (young) |
| A | 0.60 | 1.0849 | 0.05 | -1.6046 | 2.8098 | 0.56 | 0.5823 | Fall *Araucaria laubenfelsii* |
| B | 30.83 | 0.9891 | 0.05 | 28.8223 | 32.8471 | 31.17 | <.0001 | Fall *Araucaria laubenfelsii* |
| c | 0.06 | 0.00467 | 0.05 | 0.0541 | 0.0731 | 13.63 | <.0001 | Fall *Araucaria laubenfelsii* |
| A | 2.26 | 1.0542 | 0.05 | 0.1175 | 4.4071 | 2.15 | 0.0393 | Fall *Araucaria columnaris* |
| B | 33.67 | 0.9461 | 0.05 | 31.7489 | 35.5986 | 35.59 | <.0001 | Fall *Araucaria columnaris* |
| c | 0.05 | 0.00387 | 0.05 | 0.0429 | 0.0586 | 13.1 | <.0001 | Fall *Araucaria columnaris* |
| A | 0.62 | 1.3376 | 0.05 | -2.097 | 3.3456 | 0.47 | 0.6438 | Fall *Araucaria heterophylla* |
| B | 39.37 | 1.2071 | 0.05 | 36.9119 | 41.8236 | 32.61 | <.0001 | Fall *Araucaria heterophylla* |
| c | 0.06 | 0.00435 | 0.05 | 0.048 | 0.0657 | 13.08 | <.0001 | Fall *Araucaria heterophylla* |
| A | 2.95 | 1.1179 | 0.05 | 0.6724 | 5.2212 | 2.64 | 0.0127 | Fall *Araucaria bidwillii* |
| B | 24.79 | 1.0112 | 0.05 | 22.7334 | 26.8481 | 24.52 | <.0001 | Fall *Araucaria bidwillii* |
| c | 0.06 | 0.00582 | 0.05 | 0.0467 | 0.0704 | 10.05 | <.0001 | Fall *Araucaria bidwillii* |
| A | 5.73 | 0.529 | 0.05 | 4.6581 | 6.8107 | 10.84 | <.0001 | Fall *Agathis robusta* |
| B | 17.85 | 0.5298 | 0.05 | 16.7696 | 18.9252 | 33.69 | <.0001 | Fall *Agathis robusta* |
| c | 0.03 | 0.00341 | 0.05 | 0.0275 | 0.0413 | 10.1 | <.0001 | Fall *Agathis robusta* |
| A | 3.30 | 0.6805 | 0.05 | 1.9148 | 4.6838 | 4.85 | <.0001 | Fall *Agathis lanceolata* |
| B | 27.90 | 0.6109 | 0.05 | 26.6527 | 29.1386 | 45.66 | <.0001 | Fall *Agathis lanceolata* |
| c | 0.05 | 0.00298 | 0.05 | 0.0422 | 0.0543 | 16.2 | <.0001 | Fall *Agathis lanceolata* |
| A | 2.09 | 1.0656 | 0.05 | -0.0776 | 4.2584 | 1.96 | 0.0583 | Fall *Agathis australis* |
| B | 26.98 | 0.9599 | 0.05 | 25.0279 | 28.9339 | 28.11 | <.0001 | Fall *Agathis australis* |
| c | 0.06 | 0.00501 | 0.05 | 0.0452 | 0.0656 | 11.05 | <.0001 | Fall *Agathis australis* |
| A | 3.45 | 0.6982 | 0.05 | 2.034 | 4.8752 | 4.95 | <.0001 | Fall *Wollemia nobilis* (1) |
| B | 25.75 | 0.6323 | 0.05 | 24.4646 | 27.0373 | 40.73 | <.0001 | Fall *Wollemia nobilis* (1) |
| c | 0.04 | 0.00323 | 0.05 | 0.0371 | 0.0503 | 13.52 | <.0001 | Fall *Wollemia nobilis* (1) |
| A | 3.40 | 0.7506 | 0.05 | 1.8739 | 4.928 | 4.53 | <.0001 | Fall *Wollemia nobilis* (2) |
| B | 24.72 | 0.6908 | 0.05 | 23.3111 | 26.1222 | 35.78 | <.0001 | Fall *Wollemia nobilis* (2) |
| c | 0.04 | 0.00357 | 0.05 | 0.0334 | 0.0479 | 11.38 | <.0001 | Fall *Wollemia nobilis* (2) |
