# Supplementary material for: Digestibility of dinosaur food plants revisited and expanded: Previous data, new taxa, microbe donors, foliage maturity, and seasonality
Source: PLoS One. 2023 Dec 15;18(12):e0291058. doi: 10.1371/journal.pone.0291058 (PMC10723699; doi:10.1371/journal.pone.0291058)
Supplement: Attachment — Submitted filename: PONE-D-22-20691.pdf [file pone.0291058.s005.pdf]

PLOS ONE

Digestibility of dinosaur food plants revisited and expanded: Previous data, new taxa, microbe donors, foliage maturity, and seasonality

--Manuscript Draft--

**Manuscript Number:**	PONE-D-22-20691
**Article Type:**	Research Article
**Full Title:**	Digestibility of dinosaur food plants revisited and expanded: Previous data, new taxa, microbe donors, foliage maturity, and seasonality
**Short Title:**	Digestibility of dinosaur food plants revisited and expanded
**Corresponding Author:**	Mariah Howell<br>University of Bonn<br>Bonn, NRW GERMANY
**Keywords:**	dinosaur herbivory; dinosaur food plants; digestibility of the Mesozoic flora; in vitro fermentation; Hohenheim gas test; megaherbivore nutrition, dinosaur diet; Araucaria digestibility; Equisetum digestibility; Angiopteris digestibility; Araucaria fermentation; Equisetum fermentation; Angiopteris fermentation
**Abstract:**	Although once assumed to constitute a nutritionally poor diet for dinosaur herbivores, the living relatives of the Mesozoic flora and <i>in vitro</i> fermentation of their foliage have shown that gymnosperms, ferns, and fern relatives can be as highly digestible as angiosperm grasses and dicots. Because nutritional information cannot be preserved in the fossil record, this approach provides a novel way to evaluate the possible dietary needs and preferences of dinosaur megaherbivores, such as sauropods. However, few further studies have been conducted to supplement and confirm the high fermentative capacity of nonangiospermous taxa. Here we show that the living relatives of the Araucariaceae and Equistaceae are consistently highly digestible, even between taxa and when influenced by environmental and biological factors, while fern taxa are inconsistent on the family level. These results reinforce previous findings about the high energetic potential of Jurassic-age plant families. Using the <i>in vitro</i> Hohenheim gas test, a standardized method for assessing the metabolizable energy content of feed plants for ruminants by measuring the volume of gas produced during microbial fermentation, 14 species of fern and gymnosperm foliage from five Jurassic families were collected in the spring and fall and analyzed for their digestibility. The season in which specimens were collected was found to have to a significant effect on gas production in 12 out of 16 samples. <i>Equisetum</i>, <i>Araucaria</i>, and <i>Angiopteris</i> were the most digestible genera in both seasons, while <i>Agathis</i>, <i>Wollemia</i>, and <i>Marattia</i> were the least digestible. Furthermore, leaf maturity influences digestibility in <i>Marattia attenuata</i>, yet not in <i>Cyathea cooperi</i>. Finally, the species of the rumen fluid donor did not influence digestibility. With the original dataset supplemented by one new genus and four species, this study confirms and supplements previous results about the nutritional capacity of the living relatives of the Jurassic flora.
**Order of Authors:**	Mariah M. Howell
	Carole T. Gee
	Christian Böttger
	Karl-Heinz Südekum
**Additional Information:**	
**Question**	**Response**
**Financial Disclosure**<br><br>Enter a financial disclosure statement that describes the sources of funding for the work included in this submission. Review	The authors received no specific funding for this work.

Powered by Editorial Manager® and ProduXion Manager® from Aries Systems Corporation

the submission guidelines for detailed requirements. View published research articles from *PLOS ONE* for specific examples.<br><br>This statement is required for submission and **will appear in the published article** if the submission is accepted. Please make sure it is accurate.<br><br>**Unfunded studies**<br>Enter: *The author(s) received no specific funding for this work.*<br><br>**Funded studies**<br>Enter a statement with the following details:<br>• Initials of the authors who received each award<br>• Grant numbers awarded to each author<br>• The full name of each funder<br>• URL of each funder website<br>• Did the sponsors or funders play any role in the study design, data collection and analysis, decision to publish, or preparation of the manuscript?<br>• **NO** - Include this sentence at the end of your statement: *The funders had no role in study design, data collection and analysis, decision to publish, or preparation of the manuscript.*<br>• **YES** - Specify the role(s) played.<br><br>* typeset	
**Competing Interests**<br><br>Use the instructions below to enter a competing interest statement for this submission. On behalf of all authors, disclose any competing interests that could be perceived to bias this work—acknowledging all financial support and any other relevant financial or non-financial competing interests.<br><br>This statement is required for submission and **will appear in the published article** if the submission is accepted. Please make sure it is accurate and that any funding sources listed in your Funding Information later in the submission form are also	The authors have declared that no competing interests exist.

declared in your Financial Disclosure statement.<br><br>View published research articles from *PLOS ONE* for specific examples.<br><br>**NO authors have competing interests**<br><br>Enter: *The authors have declared that no competing interests exist.*<br><br>**Authors with competing interests**<br><br>Enter competing interest details beginning with this statement:<br><br>*I have read the journal's policy and the authors of this manuscript have the following competing interests: [insert competing interests here]*<br><br>* typeset	
**Ethics Statement**<br><br>Enter an ethics statement for this submission. This statement is required if the study involved:<br><br>• Human participants<br>• Human specimens or tissue<br>• Vertebrate animals or cephalopods<br>• Vertebrate embryos or tissues<br>• Field research<br><br>Write "N/A" if the submission does not require an ethics statement.<br><br>General guidance is provided below. Consult the submission guidelines for detailed instructions. **Make sure that all information entered here is included in the Methods section of the manuscript.**	The animals used for obtaining liquid ruminal content were kept in strict accordance with the German Animal Welfare legislation. All experimental procedures were approved in advance by the Animal Care Committee of the state of North Rhine-Westphalia in Germany (File Number 84-02.04.2017.A247 [cattle] and 81-02.05.40.18.008 [sheep]).

Powered by Editorial Manager® and ProduXion Manager® from Aries Systems Corporation

**Format for specific study types**<br><br>**Human Subject Research (involving human participants and/or tissue)**<br>• Give the name of the institutional review board or ethics committee that approved the study<br>• Include the approval number and/or a statement indicating approval of this research<br>• Indicate the form of consent obtained (written/oral) or the reason that consent was not obtained (e.g. the data were analyzed anonymously)<br><br>**Animal Research (involving vertebrate animals, embryos or tissues)**<br>• Provide the name of the Institutional Animal Care and Use Committee (IACUC) or other relevant ethics board that reviewed the study protocol, and indicate whether they approved this research or granted a formal waiver of ethical approval<br>• Include an approval number if one was obtained<br>• If the study involved *non-human primates*, add *additional details* about animal welfare and steps taken to ameliorate suffering<br>• If anesthesia, euthanasia, or any kind of animal sacrifice is part of the study, include briefly which substances and/or methods were applied<br><br>**Field Research**<br><br>Include the following details if this study involves the collection of plant, animal, or other materials from a natural setting:<br>• Field permit number<br>• Name of the institution or relevant body that granted permission	
**Data Availability**<br><br>Authors are required to make all data underlying the findings described fully available, without restriction, and from the time of publication. PLOS allows rare exceptions to address legal and ethical concerns. See the PLOS Data Policy and FAQ for detailed information.	Yes - all data are fully available without restriction

A Data Availability Statement describing where the data can be found is required at submission. Your answers to this question constitute the Data Availability Statement and **will be published in the article**, if accepted.<br><br>**Important:** Stating 'data available on request from the author' is not sufficient. If your data are only available upon request, select 'No' for the first question and explain your exceptional situation in the text box.<br><br>Do the authors confirm that all data underlying the findings described in their manuscript are fully available without restriction?	
**Describe where the data may be found in full sentences. If you are copying our sample text, replace any instances of XXX with the appropriate details.**<br><br>• If the data are **held or will be held in a public repository**, include URLs, accession numbers or DOIs. If this information will only be available after acceptance, indicate this by ticking the box below. For example: *All XXX files are available from the XXX database (accession number(s) XXX, XXX.*).<br>• If the data are all contained **within the manuscript and/or Supporting Information files**, enter the following: *All relevant data are within the manuscript and its Supporting Information files.*<br>• If neither of these applies but you are able to provide **details of access elsewhere**, with or without limitations, please do so. For example:<br><br>*Data cannot be shared publicly because of [XXX]. Data are available from the XXX Institutional Data Access / Ethics Committee (contact via XXX) for researchers who meet the criteria for access to confidential data.*<br><br>*The data underlying the results presented in the study are available from (include the name of the third party*	All relevant data are within the manuscript and its Supporting Information files.

and contact information or URL).<br>• This text is appropriate if the data are owned by a third party and authors do not have permission to share the data.<br><br>* typeset	
Additional data availability information:	

Manuscript

Click here to access/download;Manuscript;Howell et al._Manuscript.docx

Digestibility of dinosaur food plants revisited and expanded: Previous data, new taxa, microbe donors, foliage maturity, and seasonality

Mariah M. Howell[1*], Carole T. Gee[1,2], Christian Böttger[3], Karl-Heinz Südekum[3]

[1]Institute of Geosciences, Division of Paleontology, University of Bonn, Bonn, Germany

[2]Huntington Botanical Gardens, San Marino, Californa, United States of America

[3]Institute of Animal Science, University of Bonn, Bonn, Germany

*corresponding author

E-mail: mariah.howell@uni-bonn.de (MMH)

Abstract

Although once assumed to constitute a nutritionally poor diet for dinosaur herbivores, the living relatives of the Mesozoic flora and *in vitro* fermentation of their foliage have shown that gymnosperms, ferns, and fern relatives can be as highly digestible as angiosperm grasses and dicots. Because nutritional information cannot be preserved in the fossil record, this approach provides a novel way to evaluate the possible dietary needs and preferences of dinosaur megaherbivores, such as sauropods. However, few further studies have been conducted to supplement and confirm the high fermentative capacity of nonangiospermous taxa. Here we show that the living relatives of the Araucariaceae and Equistaceae are consistently highly digestible, even between taxa and when influenced by environmental and biological factors, while fern taxa are inconsistent on the family level. These results reinforce previous findings about the high energetic potential of Jurassic-age plant families. Using the *in vitro* Hohenheim gas test, a standardized method for assessing the metabolizable energy content of feed plants for ruminants by measuring the volume of gas produced during microbial fermentation, 14 species of fern and gymnosperm foliage from five Jurassic families were collected in the spring and fall and analyzed for their digestibility. The season in which specimens were collected was found to have to a significant effect on gas production in 12 out of 16 samples. *Equisetum*, *Araucaria*, and *Angiopteris* were the most digestible genera in both seasons, while *Agathis*, *Wollemia*, and *Marattia* were the least digestible. Furthermore, leaf maturity influences digestibility in *Marattia attenuata*, yet not in *Cyathea cooperi*. Finally, the species of the rumen fluid donor did not influence digestibility. With the original dataset supplemented by one new genus and four species, this study confirms and supplements previous results about the nutritional capacity of the living relatives of the Jurassic flora.

Introduction

Although novel research has shown that some living relatives of the Mesozoic flora have the capacity to be as highly digestible and energetically nutritious for herbivores as grasses [1], which refuted the hypothesis that nonangiospermous plants are nutritionally poor compared to flowering plants, little additional research has been conducted since to confirm and further understand the digestive properties of Jurassic-age plant families. From the Jurassic to Early Cretaceous, the flora was dominated by a wide variety of ferns, horsetails, and gymnosperms such as cycads, bennettititaleans, ginkgoes, and conifers such as the Cheirolepidiaceae and Araucariaceae [2]. Angiosperms did not appear until the Early Cretaceous, despite dominating modern ecosystems and herbivorous diets [3]; most of the phylogenetic and ecological radiation of the angiosperms occurred in the Late Cretaceous [4]. As such, the diets of herbivorous dinosaurs, including sauropods, would have been comprised of gymnosperms, ferns, and horsetails for much of the Mesozoic. Determining the digestive properties of the Jurassic flora would therefore be key to understanding the feeding ecology of these megaherbivores. However, this information cannot be preserved through fossilization, and these nonangiospermous taxa were suspected to be poorly digestible compared to flowering plants for many years. The reconstruction of sauropod diets was driven by the question of how such a nutritionally poor diet influenced the evolution and biology of the largest land animals to ever live [5–8].

In a pivotal study, Hummel et al. [1] performed an *in vitro* digestive experiment, the Hohenheim gas test, to examine the digestibility and energetic yield of the living relatives of the Mesozoic flora. The Hohenheim gas test is a well-known, standardized laboratory method that analyzes the digestibility of livestock feed by fermenting plant matter in the rumen liquid of cows, sheep, or other ruminants. This method allows the intestinal microflora to break down the

digestible fiber in the plant matter in the lab, producing gas [9, 10]. The amount of gas produced directly correlates to the digestibility of the plant matter and, therefore, the amount of metabolizable energy that an herbivore can extract from any given plant [11], as opposed to the ash test that was used by Weaver [5], which quantified the caloric value of both the digestible and indigestible fractions [1]. To approximate the digestibility of ancient plants by applying the Hohenheim gas test to the nearest living relatives of Jurassic plants, it is possible to compare the digestibility and relative amount of energy that dinosaurs may have been able to extract from the nonangiospermous flora of the Mesozoic. While not all lineages—for instance, the Bennettitales, Cheirolepidiaceae, or Pteridospermales (seed ferns)—have living members that can be tested with this method, a number of Jurassic families have survived until today. These include the Equisetaceae, Araucariaceae, Cyatheaceae, Marattiaceae, and Osmundaceae.

With this approach, Hummel et al. [1] determined that many of these living relatives, particularly those of the Equisetaceae and Araucariaceae, equaled or even surpassed angiosperm grasses and dicot leaves in digestibility and energy yield. Other species, such as ferns in the Marattiaceae and Osmundaceae, were also nearly as digestible as grasses, while *Ginkgo* was shown to be as digestible as dicot browse while also providing as much crude protein as grasses. Later, Gill et al. [12] used the Hohenheim gas test and found similar, or even greater, digestibility in plants grown under experimentally increased CO_2 levels that mimicked Mesozoic atmospheric conditions, which had been hypothesized to decrease the nutritional quality of the Mesozoic flora [7, 8]. Concrete data on the nutrition and digestibility of Jurassic plant families also allowed for estimations of daily food intake tailored to different dietary compositions [1, 12]. However, in spite of the novel approach of this method, there has been no additional research to substantiate the findings and follow up with a larger survey of taxa.

The present study quantifies the digestibility of the nearest living relatives of five Jurassic-age families using the Hohenheim gas test, confirming the dataset from previous research and supplementing it with additional species and analyses. For example, *Wollemia* is the last of the three genera in the Araucariaceae to be analyzed and completes the experimentation on the family, and two previously unanalyzed species were included in the genus *Agathis*, increasing the number of *Agathis* species tested from one to three. Among the ferns, the existing data on the Marattiaceae, which was previously represented only by *Angiopteris evecta*, was also expanded with the inclusion of a second genus, *Marattia*. Furthermore, multiple factors that affect digestibility, such as microbe donor species, foliage maturity, and seasonality, are also analyzed.

Materials and methods

Sample collection

Samples were collected from 14 species representing the nearest living relatives of Mesozoic flora from the families of the Equisetaceae (2 species), Marattiaceae (2 species), Osmundaceae (1 species), Cyatheaceae (1 species), and Araucariaceae (8 species), as well as the marine angiosperm family Zosteraceae (1 species) (Table 1). In the case of the Equisetaceae, above-ground parts were harvested, while leaf-bearing twigs were collected from the Araucariaceae. Individual fronds were taken from the Marattiaceae, Osmundaceae, and Cyatheaceae. In two species, *Marattia attenuata* and *Cyathea cooperi*, a distinction was made between “older” and “younger” leaves. Two clumps of leaves were gathered from the Zosteraceae species *Zostera marina* during low tide.

All specimens were collected from the Huntington Library, Art Collections, and Botanical Gardens in San Marino, California, USA, except for *Zostera marina* (Zosteraceae),

which was collected 50 m north of La Jolla Pier, California. Sampling initially occurred in April 2018 (hereafter called "spring"), then a second collection was gathered from the same individual plants in September 2018 ("fall"). *Zostera marina* was collected in spring but was not collected again in the fall. Specimens were dried for 48 hours at 60°C as soon as possible after collection. Four specimens were not completely dried and were heated at 60°C for an additional three hours (spec. nos. 18-28-3; 18-28-4; 18-28-7; 18-28-8). The dried samples were then milled successively using 3 mm and 1 mm sieves using a Retsch SM1 cutting mill (Haan, Germany).

Table 1. Specimens collected and evaluated using the Hohenheim gas test.

Collection Date	Spec. No.	Species	Family	Notes
4/7/2018	18-28-1	*Zostera marina*	Zosteraceae	2 clumps from beach, low
4/9/2018	18-28-3	*Angiopteris evecta*	Marattiaceae	Frond 1
4/9/2018	18-28-4	*Angiopteris evecta*	Marattiaceae	Frond 2
4/9/2018	18-28-5	*Equisetum*	Equisetaceae	
4/9/2018	18-28-6	*Osmunda regalis*	Osmundaceae	Access. no. 87695*1
4/9/2018	18-28-7	*Marattia attenuata*	Marattiaceae	Young leaf
4/9/2018	18-28-8	*Marattia attenuata*	Marattiaceae	Older leaf, same individual
4/9/2018	18-28-9	*Equisetum hyemale*	Equisetaceae	
4/9/2018	18-28-10	*Cyathea cooperi*	Cyatheaceae	Older leaf
4/9/2018	18-28-11	*Cyathea cooperi*	Cyatheaceae	Younger leaf, same
4/9/2018	18-28-12	*Araucaria*	Araucariaceae	
4/9/2018	18-28-13	*Araucaria*	Araucariaceae	Access. no. 33438
4/9/2018	18-28-14	*Araucaria*	Araucariaceae	Access. no. 32460
4/9/2018	18-28-15	*Araucaria bidwillii*	Araucariaceae	Access. no. 87754
4/9/2018	18-28-16	*Agathis robusta*	Araucariaceae	Access. no. 18450
4/9/2018	18-28-17	*Agathis lanceolata*	Araucariaceae	Access. no. 71561
4/9/2018	18-28-18	*Agathis australis*	Araucariaceae	Access. no. 71261
4/9/2018	18-28-19	*Wollemia nobilis*	Araucariaceae	Access. no. 95020*10
4/9/2018	18-28-20	*Wollemia nobilis*	Araucariaceae	Access. no. 95020*9
9/10/2018	18-28-21	*Angiopteris evecta*	Marattiaceae	Frond 1, low light
9/10/2018	18-28-22	*Angiopteris evecta*	Marattiaceae	Frond 2, direct light
9/10/2018	18-28-23	*Equisetum*	Equisetaceae	
9/10/2018	18-28-24	*Osmunda regalis*	Osmundaceae	Access no. 87695*1
9/10/2018	18-28-25	*Marattia attenuata*	Marattiaceae	Young leaf
9/10/2018	18-28-26	*Marattia attenuata*	Marattiaceae	Older leaf, same individual

9/10/2018	18-28-27	*Equisetum hyemale*	Equisetaceae	
9/10/2018	18-28-28	*Cyathea cooperi*	Cyatheaceae	Older leaf
9/10/2018	18-28-29	*Cyathea cooperi*	Cyatheaceae	Younger leaf, same
9/10/2018	18-28-30	*Araucaria*	Araucariaceae	
9/10/2018	18-28-31	*Araucaria*	Araucariaceae	Access. no. 33438
9/10/2018	18-28-32	*Araucaria*	Araucariaceae	Access. no. 32460
9/10/2018	18-28-33	*Araucaria bidwillii*	Araucariaceae	Access. no. 87754
9/10/2018	18-28-34	*Agathis robusta*	Araucariaceae	Access. no. 18450
9/10/2018	18-28-35	*Agathis lanceolata*	Araucariaceae	Access. no. 71561
9/10/2018	18-28-36	*Agathis australis*	Araucariaceae	Access. no. 71261
9/10/2018	18-28-37	*Wollemia nobilis*	Araucariaceae	Access. no. 95020*10
9/10/2018	18-28-38	*Wollemia nobilis*	Araucariaceae	Access. no. 95020*9

Gas production

The digestibility of each species was determined using a modified version of the *in vitro* Hohenheim gas test [9, 10] at the Institute of Animal Science, University of Bonn, Germany. Each sample was tested in four trials, with the exception of the spring *Osmunda regalis* (spec. no. 18-28-6) and young spring *Marattia attenuata* (spec. no. 18-28-7), which were each tested twice due to low sample volume. One sample, the fall leaves of *Araucaria heterophylla* "*glauca*" (spec. no. 18-26-33), leaked during the third trial and was retested at a later time with two replicants, for a total of five complete data sets.

The milled plant material (200 mg ± 10 mg) was inserted along with rumen fluid containing fermentative microbes and a buffer solution into sealed syringes. Rumen fluid was collected from either ruminally cannulated cattle (steer; trials 1 and 2) or ruminally cannulated sheep (trials 3 and 4) fed a standardized diet (hay and concentrate; 70:30) according to the animal maintenance requirements. The animals used for obtaining liquid ruminal content were kept in strict accordance with the German Animal Welfare legislation. All experimental procedures were approved in advance by the Animal Care Committee of the state of North

Rhine-Westphalia in Germany (File Number 84-02.04.2017.A247 [cattle] and 81-02.05.40.18.008 [sheep]).

The buffer solution was prepared according to the mixture outlined by Menke and Steingaß [10] but modified to the concentration recommendations of Liu et al. [13] to accommodate the long incubation period. Three blanks containing rumen fluid–buffer solution without added samples, three hay standard samples, and three concentrate standard samples were included in each trial to allow for calibration of results. The hay standard and concentrate samples were obtained from the Department of Animal Nutrition at Hohenheim University in Stuttgart, Germany. Each sample was incubated in a rotating syringe rack at 39°C for 96 hours to allow for maximum potential digestion, thereby removing time as a limiting factor. Gas volume, in milliliters, was recorded at the 4, 8, 12, 24, 32, 48, 56, 72, and 96-hour marks and subsequently corrected for blank gas production and expressed as ml/200 mg plant dry matter (DM).

Mathematical and statistical analysis

The parameters for gas production were fitted by a non-linear regression using PROC NLIN in SAS [14], and the following model was used to plot the kinetics of gas production in Excel [15]:

$$Gp = a + b(1 - e^{-ct})$$

wherein *Gp* represents total gas production (ml/200 mg DM), $a + b$ is the maximum gas production (ml/200 mg DM), *a* is the initial gas production of the rapidly digestible fraction (ml/200 mg DM), *b* is the gas production of the remaining slowly digestible fraction (ml/200 mg DM), *c* is the rate of gas production (h^{-1}), and *t* is time (h) [16, 17]. Significance of analyzed

variables was determined using the Wilcoxon test and the Mann-Whitney *U*-test. Results were plotted in Excel [15].

Repository

The milled plant material from this experiment is archived in the collection of the Institute of Animal Science, at the University of Bonn, Germany.

Results

Gas production and seasonal change varied greatly between species as well as within family, genera, and species. The Equisetaceae were by far the most fermentatively productive family, followed by the Osmundaceae, Araucariaceae, Cyatheaceae, and finally the Marattiaceae, with each of the latter families producing similar, slowly building kinetic curves, in contrast to the rapid and high initial production in the Equisetaceae which is then followed by a plateau (Fig 1).

Fig 1. Mean gas production by family for both seasons. The quantity in parentheses indicate the number of species tested in each family.

On the species level, *Equisetum hyemale* was found to be the most digestible species in both seasons, while the mature leaves of *Marattia attenuata* were the least digestible in both collection periods (Fig 2, A and B). In 12 of 16 samples, there is a significant change ($P < 0.05$) in maximum gas production (a + b) between the two collection periods (Tables 2 and 3).

Fig 2. Gas production kinetics by season. (A) Cumulative gas production of species collected in the spring (April 2018). (B) Cumulative gas production of species collected in the fall (September 2018).

Table 2. Parameters for the fermentation of samples collected in the spring (April 2018).

Species	Cumulative gas production (ml/200 mg dry matter)										
	4 h	8 h	12 h	24 h	32 h	48 h	56 h	72 h	96 h	$a + b$	c
Angiopteris evecta	13.3	22.4	28.6	37.7	40.0	41.6	41.8	42.0	42.0	42.0	0.095
Angiopteris evecta	11.2	19.8	25.5	33.7	35.7	37.0	37.2	37.3	37.3	37.3	0.099
Equisetum giganteum	8.2	20.4	29.2	43.9	48.2	51.8	52.4	53.0	53.1	53.2	0.079
Osmunda regalis	9.3	14.8	19.3	28.9	32.9	37.5	38.9	40.4	41.4	41.9	0.056
Marattia attenuata (younger)	7.4	9.8	12.0	17.2	19.9	23.7	25.1	27.0	28.7	30.5	0.038
Marattia attenuata (older)	4.8	5.9	7.0	9.7	11.2	13.7	14.7	16.4	18.1	21.6	0.017
Equisetum hyemale	11.2	26.7	38.3	58.1	64.3	69.7	70.8	71.7	72.0	72.1	0.074
Cyathea cooperi (younger)	10.0	13.0	15.6	21.1	23.6	26.6	27.5	28.6	29.3	29.8	0.041
Cyathea cooperi (older)	7.6	11.8	15.5	23.4	26.9	31.7	32.6	34.2	35.3	35.9	0.041
Araucaria laubenfelsii	5.9	11.7	16.5	25.7	29.3	33.0	34.0	35.0	35.5	35.7	0.055
Araucaria columnaris	9.2	15.0	19.7	28.9	32.5	36.2	37.2	38.2	38.7	38.9	0.055
Araucaria heterophylla "*glauca*"	10.6	15.7	20.1	29.3	33.2	37.8	39.2	40.8	41.8	42.3	0.045
Araucaria bidwillii	9.6	15.7	20.5	29.3	32.5	35.6	36.3	37.0	37.3	37.4	0.062
Agathis robusta	9.3	11.5	13.4	18.1	20.5	23.8	25.0	26.7	28.1	29.5	0.029
Agathis lanceolata	12.7	16.8	20.3	27.8	31.1	35.2	36.4	37.9	38.9	39.5	0.041
Agathis australis	9.7	13.7	16.6	23.0	25.5	28.4	29.1	30.0	30.5	30.7	0.050
Wollemia nobilis	7.5	10.7	13.5	19.6	22.2	25.5	26.5	27.7	28.5	29.0	0.042
Wollemia nobilis	7.3	11.3	14.7	22.2	25.6	29.7	31.0	32.6	33.7	34.3	0.040

For each sample, cumulative gas production (ml/200 mg dry matter) up to 96 h and fermentative parameters, in which $a + b$ is maximum gas production (ml/200 mg dry matter) and c is rate of gas production (h^{-1}), are given.

Table 3. Parameters for the fermentation of samples collected in the fall (September 2018).

Species	Cumulative gas production (ml/200 mg dry matter)										
	4 h	8 h	12 h	24 h	32 h	48 h	56 h	72 h	96 h	*a* + *b*	*c*
Angiopteris evecta	14.1	23.0	28.7	36.1	37.7	38.6	38.7	38.7	38.8	38.8	0.112
Angiopteris evecta	16.6	25.6	31.9	41.3	43.7	45.4	45.6	45.8	45.8	45.9	0.093
Equisetum giganteum	10.6	23.8	33.2	47.6	51.5	54.5	54.9	55.3	55.4	55.42	0.087
Osmunda regalis	8.0	11.4	14.3	20.3	22.9	26.0	26.9	28.0	28.7	29.1	0.044
Marattia attenuata[1]	7.5	9.9	12.0	17.1	19.6	23.2	24.4	26.2	27.7	29.2	0.029
Marattia attenuata[2]	4.8	5.4	6.0	7.6	8.4	9.8	10.4	11.3	12.3	14.23	0.017
Equisetum hyemale	9.9	22.9	33.0	51.3	57.6	63.5	64.8	66.0	66.5	66.6	0.055
Cyathea cooperi[1]	10.0	14.2	17.6	24.7	27.5	30.8	31.6	32.6	33.2	33.5	0.047
Cyathea cooperi[2]	9.3	13.3	16.6	23.4	26.2	29.4	30.3	31.4	32.0	32.3	0.049
Araucaria laubenfelsii	7.5	12.9	17.1	24.7	27.4	30.0	30.6	31.1	31.4	31.4	0.064
Araucaria columnaris	8.4	13.5	17.6	26.0	29.3	33.0	34.0	35.1	35.7	35.9	0.051
Araucaria heterophylla "*glauca*"	8.6	15.0	20.1	30.0	33.6	37.4	38.4	39.3	39.8	40.00	0.057
Araucaria bidwillii	8.2	12.0	15.1	21.1	23.3	25.6	26.2	26.8	27.1	27.7	0.057
Agathis robusta	8.0	10.0	11.8	15.8	17.7	20.2	21.0	22.1	22.9	23.6	0.034
Agathis lanceolata	8.2	12.2	15.6	22.4	25.2	28.4	29.3	30.3	30.9	31.2	0.048
Agathis australis	7.5	11.8	15.2	21.9	24.5	27.2	27.9	28.6	28.9	29.1	0.055
Wollemia nobilis	7.6	11.1	14.0	20.2	22.9	26.0	27.0	28.1	28.8	29.2	0.044
Wollemia nobilis	7.1	10.3	13.0	18.8	21.4	24.6	25.6	26.8	27.6	28.1	0.041

For each sample, cumulative gas production (ml/200 mg dry matter) up to 96 h and fermentative parameters, in which $a + b$ is maximum gas production (ml/200 mg dry matter) and c is rate of gas production (h^{-1}), are given.

In all but three specimens, those collected in the spring yielded more gas than the same individuals did in the fall collection (Fig 3). However, while it may affect maximum gas production, seasonality was found to have no significant influence on the rate of gas production (*P* = 0.075; Wilcoxon Signed-Rank Test).

Fig 3. Mean gas production by season. Blue bars represent the maximum gas production ($a + b$) of samples collected during the spring (April 2018). Red bars the maximum gas production ($a + b$) of samples collected in the fall (September 2018). Asterisk indicates $P < 0.05$ for seasonal affects within the species (Wilcoxon test).

Ferns and fern allies were variable in their maximum gas production, rate of gas production, and seasonal effects. Rate of gas production (*c*) of both *Equisetum* species and *Angiopteris evecta* was comparatively high in both seasons (Tables 2 and 3), with rapid initial production that leveled off soon after the first 24 hours; at least 79% of gas production took place in the first 24 hours, with *A. evecta* reaching over 90% of gas production during that time (Fig 4), making it the most rapidly digestible of any species in both seasons (Table 2 and 3). Conversely, the leaves of *Marattia attenuata* had low rates of gas production and achieved 60% or less of its 96-hour production within the first 24 hours (Tables 2 and 3; Fig 4).

Fig 4. Digestion time. Percentage of maximum gas production achieved within the first 24 hours (blue) and the subsequent 72 hours (orange), for a total of 96 hours of digestion.

In terms of maximum gas production, both species of *Equisetum* outperformed all other species, with the spring samples of *E. hyemale* overtaking *E. giganteum* immediately, but the fall samples only after a lead of twelve hours. In the spring, *Osmunda regalis* was the most digestible fern despite its slower rate of production, but in the fall, *Angiopteris evecta* and both samples of *Cyathea cooperi* were more digestible. *Marattia attenuata* was the least digestible fern,

regardless of leaf maturity or season (Fig 2, A and B). However, leaf maturity still significantly affected digestibility in *Marattia attenuata* (Fig 5A; $P < 0.05$; Wilcoxon test) but did not affect the digestibility of *Cyathea cooperi* (Fig 5B; $P > 0.2$; Wilcoxon test).

Of the eight ferns and fern allies tested, five were influenced by seasonality, including both species of *Equisetum* and *Angiopteris evecta* (Fig 3). Within the same individual organism of *Marattia attenuata*, the maximum gas production of younger leaves was not significantly influenced by seasonality, but the production of older leaves was. This was not the case in *Cyathea cooperi*, in which neither the younger nor the older leaves experienced significant seasonal alterations in digestibility. *Osmunda regalis* experienced the greatest seasonal shift in gas production volume (Tables 2 and 3; Fig 3) of any species, producing 44% more gas in the spring than the fall.

Fig 5. Influence of leaf maturity on the fermentation of two species. (A) Fermentative behavior of younger and older leaflets of *Cyathea cooperi* ($P > 0.2$; Wilcoxon test). (B) Fermentative behavior of younger and older leaflets of *Marattia attenuata* ($P < 0.05$; Wilcoxon test).

In general, the Araucariaceae had lower rates of gas production than many of the ferns but achieved comparable maximum gas production (Tables 2 and 3). Members of this family tended towards slower production curves and did not begin to plateau until between 72 and 96 hours (Fig 6A). For example, in both seasons, the most digestible araucarian species was *Araucaria heterophylla* "*glauca*," which produced similar gas volumes as the ferns *Osmunda regalis* and *Angiopteris evecta* after 56 hours of digestion, in spite of its much slower initial production and more gradual digestion curve (Fig 2, A and B). The most productive genus was *Araucaria*, while *Agathis* and *Wollemia* achieved similar gas production curves (Fig 6B). However, generic affinity was not necessarily a predictor of digestibility; in the spring, *Agathis*

lanceolata was the second most digestible species in the family, while *Agathis robusta* was the least digestible (Fig 6A). *Agathis robusta* was also particularly slow to ferment, with less than 60% of its gas production occurring within the first 24 hours (Fig 4) and the lowest rate of production of any araucarian conifer in either season (Tables 2 and 3). Among the Araucariaceae, the maximum gas production of six of the eight species tested were found to be significantly influenced by season, including *Wollemia nobilis* and all species tested of *Agathis*. Only *Araucaria laubenfelsii* and *Araucaria heterophylla* "*glauca*" did not experience a seasonal change in digestibility (Fig 3).

Fig 6. Gas production of the Araucariaceae. (A) Mean gas production of araucariaceous species, plotted as individual curves. (B) Mean gas production of araucariaceous genera, plotted as averages of all species tested.

The marine angiosperm *Zostera marina*, known as common eelgrass, is excluded here from data interpretation because its cumulative gas production was too poor to be fitted to the mathematical model. After 24 hours, eelgrass produced only 4.1 ml/200 mg DM of gas, and only 10.2 ml/200 mg DM of gas after 96 hours. Additionally, the rumen fluid donor species did not have a significant influence on maximum gas production ($P = 0.872$; Mann-Whitney U-test), particularly after the first 24 hours of production.

Discussion

The living relatives of the Mesozoic flora tested here display a high capacity for digestibility and energetic yield within certain taxa. In particular, the Equisetaceae and the Araucariaceae consistently yield high quantities of energy, indicating a high potential for this family to have been a targeted food source of herbivorous dinosaurs. However, other families show greater digestive variation between species, making family-level generalizations of their

dietary usefulness difficult; while the family Marattiaceae contains the fern species yielding the lowest energy, *Marattia attenuata*, it also has the fern species with the highest energy yield, *Angiopteris evecta* (Fig 2, A and B). These results are consistent with, and even exceed, those previously found by Hummel et al. [1].

Digestibility of *Equisetum*

Equisetum, as previously shown by Hummel et al. [1] and Gill et al. [12], continues to outperform all other groups in energy yield, and in the current study, some of the highest gas production values yet for the genus have been achieved. For example, *E. hyemale* has a spring maximum gas production volume of 72.1 ml/200 mg DM (Table 2), compared to the 59.92 ml/200 mg DM in tests found in 2004 (Table A in S1 Table). The maximum gas production values for this species are marginally exceeded only by *E. hyemale* grown by Gill et al. [12] in 1200 ppm atmospheric CO_2 conditions, which reached 72.4 ml/200 mg DM. However, Gill et al. [12] determined that *E. hyemale* was not significantly affected by CO_2 concentration, which is further supported by our samples which grew outdoors in ambient CO_2 conditions yet achieved markedly similar digestibility. The consistency of these findings may also suggest that our results approach the upper digestibility potential of *E. hyemale*.

In *E. giganteum* (Tables 2 and 3), both the fall and spring gas production were higher than those of the 2004 samples (Fig B in S1 Fig; Table A in S1 Table), but the range was not as great as that in *E. hyemale.* Additional analyses on the digestibility of this genus in 2011 showed maximum gas production values between 56.6 and 61.7 ml/200 mg DM for multiple species and cultivars, including *E. arvense*, *E.* x *trachyodon*, *E.* x *moorei*, and *E. ramosissimum* (Fig B in S2 Fig; Table B in S1 Table), all of which exceed the average maximum gas production of both grasses and dicots determined by Hummel et al. [18]. However, in the 2004 experiments, *E.*

giganteum and *E. arvense* fell just short of the maximum production of grasses, at 47.3 and 49.0 ml/200mg DM of gas, respectively (Fig B in S1 Fig; Table A in S1 Table). The overall high performance across all three datasets reiterates that even with its high silica content [cf. 19], which is thought to inhibit the digestibility of foliage [20], *Equisetum* would have been a consistently nutritious option for herbivorous dinosaurs. While both *E. hyemale* and *E. giganteum* exhibit a reduction in digestibility in the fall, they are still more digestible than any other sample (Fig 3), far exceeding the standards for both modern angiosperm grasses and dicot leaves determined by Hummel et al. [18]. Furthermore, the cosmopolitan distribution of *Equisetum* and its relatives during the Jurassic and Cretaceous [21, 22] would have made it commonly available to the global fauna of herbivorous dinosaurs in the Mesozoic [23].

Digestibility of ferns

Among the ferns, digestibility is more inconsistent as they tend to be affected by season, leaf maturity, and specificity. Therefore, familial affinity is not considered to be a reliable indication of species digestibility. As a whole, the family Marattiaceae performed more poorly than any other family tested (Fig 1), despite *Angiopteris evecta* being the most digestible fern species overall (Tables 2 and 3) and the third-most digestible species in the fall dataset after *Equisetum hyemale* and *E. giganteum*. *Osmunda regalis*, at its peak in the spring, is similarly highly digestible, though it experiences a large reduction in gas production in the fall. The older leaves of *Marattia attenuata* are consistently the most poorly performing foliage (Fig 2, A and B; Tables 2 and 3).

Thus, individual fern species may have been extremely nutritious forage for herbivorous dinosaurs, but this would have been greatly influenced by environmental conditions. While a broader survey of ferns could offer deeper insights into digestibility, the results produced in this

study show the wide range of energy yield that ferns may have been capable of offering. Massive, bulk-feeding megaherbivores like sauropods likely would have exploited even poorly fermenting species like *M. attenuata*, as larger animals are typically more tolerant of poor-quality diets [24-27], although whether this is the result of an actual increase in digestive efficiency due to their large size or a result of necessity has been debated [26-28].

Digestibility of the Araucariaceae

As whole, the Araucariaceae are again confirmed to be among the most energetically nutritious taxa, particularly those of the genus *Araucaria*. The evergreen foliage of the Araucariaceae would have provided a consistent source of highly caloric food to dinosaurs that could reach the leaves of tall, mature trees, regardless of local environmental conditions. Although the araucariaceous leaves are not as immediately digestible as *Equisetum* and all but two of the species tested here experienced seasonal decreases in digestibility, the Araucariaceae, which grew in both the Northern and Southern Hemispheres during the Jurassic [23], would provide a substantial amount of energy given four or more days for fermentation. Large herbivores such as sauropods likely would have been capable of exploiting the fermentative behavior of araucariaceous species. Hummel et al. [1] found that the Araucariaceae were not only more calorically nutritious than other Mesozoic families, including cycads, podocarps, and tree ferns, but were also, over time, more digestible than the dicot leaves and grasses on which most modern herbivores feed. The results of the current study confirm the high nutritional yield of these conifers, particularly those of the genus *Araucaria* (Fig 6, A and B). With the inclusion of *Wollemia*, all three genera of the Araucariaceae have now been analyzed. While the results indicate that *Wollemia* is not as digestible as those of *Araucaria*, it is comparable in its fermentative yield and kinetic behavior to *Agathis australis* (Fig 6A).

Araucaria heterophylla was the most digestible member of the Araucariaceae tested here, with a maximum gas production of 42.3 ml/200 mg DM in the spring and 40.0 ml/200 mg DM in the fall (Tables 2 and 3). This is comparable to the maximum gas production of 41.9 ml/200 mg DM found in samples collected in 2007 (Fig A in S2 Fig; Table B in S1 Table). The spring maximum gas production value for *Araucaria laubenfelsii* found here (35.7 ml/200 mg DM; Table 2) is also similar to results from previous experiments (38.3 ml/200 mg DM; Table A in S1 Table). However, maximum gas production for *Araucaria columnaris* in the present study was much lower than the values obtained in the earlier study, while *Agathis robusta* was notably higher (Tables 2, 3; Table A in S1 Table). *Araucaria bidwillii* was variable in its gas production across the three trials, with this experiment obtaining a maximum gas production between 27.7 and 37.4 ml/200 mg DM (Tables 2 and 3). In the 2007 set, *Araucaria bidwillii* had a maximum gas production as low as 24.7 ml/200 mg DM (Table B in S1 Table), though it was as high as 40.1 ml/200 mg DM in 2004 (Table A in S1 Table). These results highlight the variability of digestibility that can occur within one species, but also the relatively uniform yield that certain taxa can have, even between different individuals in variable climates, localities, and collection periods. Overall, the results of the current study confirm the high nutritional yield of these conifers, particularly those of the genus *Araucaria* (Fig 6A, B), though *Wollemia* and *Agathis* still provide more energy than other nonangiospermous taxa.

Seasonal effects on digestibility

Overall, 12 of the 16 plants tested here were found to be significantly affected by seasonality, which encompasses factors such as temperature, the amount of sunlight, day length, and precipitation received during the growing season. Of those that experienced a significant fluctuation in digestibility between the two seasons, 10 plant taxa decreased gas production from

spring to fall and two taxa increased production (Fig 3). The species that were not affected by seasonality were not related; one was the tree fern *Cyathea cooperi*, two pertained to the conifer *Araucaria*, and the last was the younger leaves of the fern *Marattia attenuata*. In both seasons, *Equisetum hyemale* and *E. giganteum* were the most highly digestible, while the older leaves of *M. attenuata* were the least digestible of all tested material. However, the relative digestibility of all other species varied between the two collection periods. For example, in spring, the most digestible fern was *Osmunda regalis*, but the more digestible fern in the fall collection was *Angiopteris evecta* (Fig 2).

Leaf maturity effects on digestibility

Another potential factor for fluctuations in digestibility is leaf maturity. Consistent with studies on the digestibility of angiosperm livestock feed plants, younger leaves are generally more nutritious [29-31]. Thus, leaf maturity may influence digestibility in ferns, as is shown in the species *Marattia attenuata* (Fig 5). However, maturity effects for *Cyathea cooperi* were found to be insignificant (Fig 5). Interestingly, maturity may interact with seasonality, as the younger leaves of *M. attenuata* were not affected by seasonality, although the older leaves were (Fig 3). This combination could result in a large variation in digestibility within this species, as the more mature leaves in the fall produced only half as much gas as the younger leaves did in the spring. Such a nutritional gradient within an individual, poorly digestible plant would favor herbivores that could be more selective about which parts of the plant they consume, allowing them to target the younger, more nutritious leaves and avoiding the largely indigestible mature leaves, particularly late in the growing season. A larger survey would need to be conducted to determine the broader relevance of leaf maturity and the degree to which it interacts with

seasonality within these nonangiospermous species, although the inconsistency of the results here may indicate that further experiments will not yield applicable results.

Donor species effects on digestibility

The donor species of rumen fluid was not found to affect gas production. Two donor species, domestic sheep and cattle, were used in this experiment, and all donors followed diets outlined for use in the Hohenheim gas test [9]. These results are consistent with those of Cone et al. [32], who showed that gas production using cow rumen fluid and sheep rumen fluid were comparable after at least 48 hours of production. This supports the concept that the species of the donor does not influence digestive capability; rather, it is the diet of the animal that most strongly influences the composition of the gut biota and therefore efficiency in digestion [18]. As such, an animal that is accustomed to feeding on the plants analyzed here would have gut flora that is even more efficient at fermenting such material than the gut flora taken from animal feeding on standardized angiosperm foliage.

Environmental considerations

Environmental factors, such as precipitation and sunlight related to seasonality, can drastically affect the nutritional yield of plants [33]. For this study, the site of collection at the Huntington Botanical Gardens in San Marino, California is classified as having a mild temperate climate with warm, dry summers [34]. Climate data from the nearby city of Pasadena document a maximum temperature of 34.4°C and a minimum temperature of 11.1°C in the period between the two collection dates in 2018. Precipitation was low, as 12.8 mm of precipitation was recorded for May and none for the period from June to September [35].

In comparison, the climate during the Jurassic was quite different. According to Sellwood and Valdes [36], the Jurassic climate was warm and equable enough that forests grew near both

poles, which experienced warm summers but cold, dark, and potentially wet winters. The mild winters at the poles would have favored migratory dinosaurs, although equatorial regions would have been more even in climate all year-round. In contrast to today's polar to tropical climate gradient, Cretaceous temperatures were up to 10°C warmer across the globe at its peak in the Cenomanian; warm-climate plants grew in the polar regions, although there is evidence of polar cooling by the Late Cretaceous [37].

Growth rings in Jurassic wood provide direct fossil evidence of Mesozoic plants responding to environmental conditions, along with providing paleoclimatological evidence for moderate seasonality in some localities. Such growth rings have been documented in the Triassic of China [38]; the Jurassic of India [39], England [40, 41], USA [42], and Mongolia [43]; and the Cretaceous of India [39], Brazil [44, 45], and Alaska [46]. In some cases, there is evidence that the trees were subjected to stress or trauma that interrupted growth for unpredictable or irregular periods of time, such as volcanism or localized drought, rather than climatic periodicity [47-49]. Interruptions and changes in the growth in response to these environmental incidents likely would have been reflected in the nutritional capacity of the edible plant parts, at least temporarily.

Herbivore physiology and behavior

Although sauropod dinosaurs were not ruminants like the donor species used in this experiment, which have a highly derived digestive system consisting of multiple stomachs to facilitate several oral processing and digestive phases, and it is unlikely that they would have been foregut fermenters [25], the *in vitro* nature of the Hohenheim gas test minimizes the involvement of the unique digestive anatomy of ruminants while still simulating the fermentative processes that would have occurred within the gut. Furthermore, data from ruminant tests can be

reliably transformed to be comparable to results derived from tests using microbes from hindgut fermenters such as horses [50]. While the specific digestive anatomy of the sauropod dinosaurs has not yet been fully determined due to a lack of soft part preservation, the incredible size of these dinosaurs would have allowed for a massive fermentation chamber capable of digesting large volumes of plant material for an extended period of time. While larger body size does not necessarily lead to increased retention times [27, 51] as once assumed [52], extended retention times would have compensated for the slow rate of digestion of plant species such as *Agathis robusta*, *Wollemia nobilis*, or *Marattia attenuata* (Fig 2, A and B), as well as for large particle sizes [51] due to the apparent lack of mastication [25, 53], gastric mills [54], or other particle size reduction mechanisms. However, it is noteworthy that even the most slowly fermenting and weakly digestible species, *Agathis robusta*, achieved at least 56% of gas production within the first 24 hours (Fig 4), although *in vivo* fermentation and digestion would likely take longer without particle size reduction. Meanwhile, quickly fermenting plants with high digestibility similar to *Equisetum spp.* and *Angiopteris evecta* (Fig 2, A and B) would have been especially important for small-bodied, young, or growing dinosaurs, that would have needed to consume a larger proportion of higher quality material for their body size [28].

For adult sauropods, Hummel et al. [1] estimates the amount of consumed dry plant matter sauropods of different body masses and metabolic behaviors would have required based on the energetic density of the plant material, suggesting that a highly digestible diet would allow for 40% less consumed plant mass than a low-quality diet, while Gill et al. [12] calculates that a 10.8 t *Diplodocus* would only need to need 23.8 kg of *Equisetum hyemale* per day, as opposed to 33.2 kg of the fern species *Polypodium vulgare*. However, if digestibility and the energy gained from consumption of those plants were to decrease during part of the year or during times of

environmental stress, it would necessitate a greater consumed volume of material or a preference for higher-quality plants to maintain the same energy intake. Conversely, an increase in digestibility would allow for the opposite: a lower consumed volume per day or a wider variety of plants of variable nutritional capacity [28].

Elephants, a modern analog for a bulk-feeding terrestrial megaherbivore, have been found to vary their dietary composition seasonally as different forms of nutrition become more or less available; this has been shown to be true in both the savanna-dwelling African elephant [55] and the tropical Asian elephant [56]. African elephants will consume a wider variety of plants during the dry season [55], whereas Asian elephants will expand or change the habitats they occupy in order to feed on more seasonally nutritional plants [57]. Additionally, while African elephants consume a larger volume of material during the wet season than the dry season, this likely results from the lower availability of resources during the dry season, which is reflected in a corresponding decrease in the African elephants' physical condition due to nutritional stress [58]. Similar seasonal correlations have been found for other megaherbivores, including black rhinoceroses [59], greater one-horned rhinoceroses [56], giraffes [60], African buffalo [61], and even extinct giant ground sloths [62].

Among birds, which have a strong phylogenetic relationship to dinosaurs, there are few folivores because of the metabolic and behavioral requirements necessary to make such a diet viable in a small-bodied organism [63]. Those that do engage in folivorous activity, however, display seasonal dietary behavioral changes. Ostriches, which have been previously used as an analog for sauropod dinosaurs to investigate digestive anatomy [54], have a strong preference for particular food species, but will greatly broaden their target species when feeding during the dry season [64]. Hoatzin, a South American, foregut fermenting species, modify their diets in

response to seasonal changes in the availability of their preferred food sources; though their diet predominantly consists of leaves, at the end of the dry season when trees lose their foliage, they supplement their diet with flowers [65]. The speckled mousebird also compensates for seasonal food limitations by tolerating a wide variety of resources [66]. Similarly, the white-tipped plantcutter feeds on a much wider variety of species during the winter and spends up to six times longer foraging during this time because of the limited availability of evergreen species [67].

Herbivorous dinosaurs may have exhibited similar dietary behavior changes to compensate for fluctuations in the digestibility of the Mesozoic flora or other biological needs. Isotopes in the tooth enamel of *Camarasaurus* suggests these sauropods migrated seasonally, shedding light on how they were able to occupy a seasonally dry habitat where they would have otherwise experienced water and nutritional stress [68]. There may be further evidence of seasonally induced dietary plasticity in dinosaurs; for example, Chin et al. [69] interprets coprolite evidence of crustacean consumption by ornithischian dinosaurs as a seasonal feeding habit, possibly in response to nutritional needs induced by reproductive cycles, suggesting that even bulk-feeding herbivorous dinosaurs engaged in targeted feeding when necessary. Particularly if they resulted from reproductive-stage nutritional needs, these behavioral responses to seasonal shifts in available nutrition may have been dependent on age and sex, as they are in modern herbivores. For example, Woolley et al. [70] reports that male African elephants tolerate lower-quality diets better than females do.

Future research

The inconsistent results of the effects of seasonality and leaf maturity may indicate that further experimentation is necessary; however, it may also indicate that these responses are species dependent and therefore uninformative to the characterization of fossil taxa. Tests on

other aspects of nutritional quality such as determination of the macronutrients and C/N ratio, as previously examined by Midgley et al. [7], Hummel et al. [1], Wilkinson and Ruxton [8], and Gill et al. [12] would provide more information about other aspects of nutrition beyond digestibility and relative caloric value. Overall, a broader survey of plants within Mesozoic-age families would be informative, particularly of the Podocarpaceae and those in the Cycadales. Available data on these families show that taxa in both groups are digestively poor, and testing a broader range of species would confirm or refute these results. Additionally, further experimentation on additional living ferns with close relatives in the Mesozoic would clarify the wide range of digestive behavior seen here in the Marattiaceae and Osmundaceae. Tests of angiosperm families with deep paleontological roots in the Cretaceous, such as the water lilies of the Nymphaeaceae or palms of the Arecaceae, would also provide a comparison for herbivory on Cretaceous angiosperms.

Conclusions

With the inclusion of one new genus and four new species, as well as the added dimensions of seasonality and leaf maturity in nonangiospermous species, the new results reported here both support and expand upon previous conclusions regarding the high potential digestibility and energy content of the nearest living relatives of the Mesozoic flora. Most notably, all species of the horsetail genus *Equisetum* continue to be the most digestible and energetically profitable, while the conifer genus *Araucaria* is similarly confirmed to be a viable, consistently high performing food resource for herbivorous dinosaurs. Although the other Araucariaceae genera, *Agathis* and *Wollemia*, were on average slightly less digestible than *Araucaria*, gas production was still comparable to that of *Araucaria*. Ferns of the Marattiaceae and Osmundaceae are more variable between and within species, with some, such as *Angiopteris*

evecta, providing consistently high nutrition, *Osmunda regalis* providing seasonably variable nutrition, and *Marattia attenuata* and *Cyathea cooperi* being more poorly digestible. Therefore, familial affinity cannot yet be used to predict the digestibility of fern species. Additionally, the influence of environmental and biological factors is inconsistent. In most species, the foliage shows significant fluctuations in digestibility in accordance with seasonality, and the relative digestibility of most taxa is dependent on season. While these fluctuations in digestibility may be further influenced by leaf maturity, they are not affected experimentally by the herbivore donor of rumen fluid. These results contribute to a more complete understanding of the nutritive capacity of Mesozoic plants for the herbivores that relied on them.

Acknowledgments

We sincerely thank Jim Folsom and Sean Lahmeyer at the Huntington Botanical Gardens for allowing and facilitating the collection of plant samples, as well as Kelly Shunn for the collection and transport of the material.

References

1. Hummel J, Gee CT, Südekum KH, Sander PM, Nogge G, Clauss M. In vitro digestibility of fern and gymnosperm foliage: Implications for sauropod feeding ecology and diet selection. Proc R Soc B Biol Sci. 2008;275(1638): 1015–1021.
2. Gee C.T. Plants in Mesozoic time: Morphological innovations, phylogeny, ecosystems. Bloomington: Indiana University Press; 2010.
3. Hofmann RR, Stewart DRM. Grazer or browser: A classification based on the stomach-structure and feeding habits of East African ruminants. Mammalia. 1972;36(1): 226–40.

4. Friis EM, Crane PR, Pedersen KR. Early flowers and angiosperm evolution. Cambridge: Cambridge University Press; 2011.
5. Weaver JC. The improbable endotherm: The energetics of the sauropod dinosaur *Brachiosaurus*. Paleobiology. 1983;9(2): 173–182.
6. Fiorillo AR. Dental microwear patterns of the sauropod dinosaurs camarasaurus and diplodocus: Evidence for resource partitioning in the Late Jurassic of North America. Hist Biol. 1998;13(1): 1–16.
7. Midgley JJ, Midgley G, Bond WJ. Why were dinosaurs so large? A food quality hypothesis. Evol Ecol Res. 2002;4(7): 1093–1095.
8. Wilkinson DM, Ruxton GD. High C/N ratio (not low-energy content) of vegetation may have driven gigantism in sauropod dinosaurs and perhaps omnivory and/or endothermy in their juveniles. Funct Ecol. 2013;27(1): 131–135.
9. Menke KH, Raab L, Salewski A, Steingass H, Fritz D, Schneider W. The estimation of the digestibility and metabolizable energy content of ruminant feedingstuffs from the gas production when they are incubated with rumen liquor in vitro. J Agric Sci. 1979;93(1): 217–22.
10. Menke KH, Steingass H. Estimation of the energetic feed value obtained from chemical analysis and in vitro gas production using rumen fluid. Anim Res Dev. 1988;28: 7–55.
11. Van Soest PJ. Allometry and ecology of feeding behavior and digestive capacity in herbivores: A review. Zoo Biol. 1996;15(5): 455–79.
12. Gill FL, Hummel J, Sharifi AR, Lee AP, Lomax BH. Diets of giants: The nutritional value of sauropod diet during the Mesozoic. Palaeontology. 2018;61(5): 647–58.

13. Liu JX, Susenbeth A, Südekum KH. In vitro gas production measurements to evaluate interactions between untreated and chemically treated rice straws, grass hay, and mulberry leaves. J Anim Sci. 2002;80(2): 517–24.

14. Institute S. SAS user's guide: Statistics. Vol. 2. SAS Inst; 2013.

15. Microsoft Corporation. Microsoft Excel. 2016. Available from: https://office.microsoft.com/excel

16. Orskov ER, McDonald I. The estimation of protein degradability in the rumen from incubation measurements weighted according to rate of passage. J Agric Sci. 1979;92(2): 499–503.

17. McDonald I. Short note: A revised model for the estimation of protein degradability in the rumen. J Agric Sci. 1981;96(1): 251–252.

18. Hummel J, Südekum KH, Streich WJ, Clauss M. Forage fermentation patterns and their implications for herbivore ingesta retention times. Funct Ecol. 2006;20(6): 989–1002.

19. Holzhüter G, Narayanan K, Gerber T. Structure of silica in *Equisetum arvense*. Anal Bioanal Chem. 2003;376(4): 512–517.

20. Van Soest PJ, Jones LHP. Effect of silica in forages upon digestibility. J Dairy Sci. 1968;51(10): 1644–1648.

21. Schaffner JH. Geographic distribution of the species of *Equisetum* in relation to their phylogeny. Am Fern J. 1930;20(3): 89–106.

22. Taylor TN, Taylor EL, Krings M. Paleobotany: The biology and evolution of fossil plants. 2nd ed. Amsterdam: Elsevier; 2009.

23. Gee CT. Dietary options for the sauropod dinosaurs from an integrated botanical and paleobotanical perspective. In: Klein N, Remes K, Gee CT, Sander PM, editors. Biology of the sauropod dinosaurs: Understanding the life of giants. Indiana University Press; 2011. pp. 34–56.

24. Bell R. A Grazing ecosystem in the Serengeti. Sci Am. 1971;225: 86–93.

25. Hummel J, Clauss M. Sauropod feeding and digestive physiology. In: Klein N, Remes K, Gee CT, Sander PM, editors. Biology of the Sauropod Dinosaurs: Understanding the Life of Giants. Indiana University Press; 2011. pp. 11–33.

26. Müller DWH, Codron D, Meloro C, Munn A, Schwarm A, Hummel J, et al. Assessing the Jarman–Bell Principle: Scaling of intake, digestibility, retention time and gut fill with body mass in mammalian herbivores. Comp Biochem Physiol Part A. 2013;164(1): 129–140.

27. Steuer P, Südekum K-H, Tütken T, Müller DWH, Kaandorp J, Bucher M, et al. Does body mass convey a digestive advantage for large herbivores? Funct Ecol. 2014;28: 1127–1134.

28. Clauss M, Steuer P, Müller DWH, Codron D, Hummel J. Herbivory and body size: Allometries of diet quality and gastrointestinal physiology, and implications for herbivore ecology and dinosaur gigantism. PLoS One. 2013;8(10): e68714.

29. Pritchard GI, Folkins LP, Pidgen WJ. The in vitro digestibility of whole grasses and their parts at progressive stages of maturity. Can J Plant Sci. 1962;43: 79–87.

30. Terry RA, Tilley JMA. The digestibility of the leaves and stems of perennial ryegrass, cocksfoot, timothy, tall fescue, lucerne and sainfoin, as measured by an in vitro procedure. Grass Forage Sci. 1964;19(4): 363–372.

31. Santos MB, Nader GA, Robinson PH, Kiran D, Krishnamoorthy U, Gomes MJ. Impact of simulated field drying on in vitro gas production and voluntary dry matter intake of rice straw. Anim Feed Sci Technol. 2010;159(3–4): 96–104.

32. Cone JW, Van Gelder AH, Bachmann H. Influence of inoculum source on gas production profiles. Anim Feed Sci Technol. 2002;99(1–4): 221–231.

33. Krizsan SJ, Nyholm L, Nousiainen J, Südekum KH, Huhtanen P. Comparison of in vitro and in situ methods in evaluation of forage digestibility in ruminants. J Anim Sci. 2012;90(9): 3162–3173.

34. Chen D, Chen HW. Using the Köppen classification to quantify climate variation and change: An example for 1901–2010. Environ Dev. 2013;6(1): 69–79.

35. Lawrimore JK, Ray R, Applequist S, Korzeniewski B, Menne MJ. Global Summary of the Month (GSOM), Version 1. GHCND:USC00046719. 2016. NOAA National Centers for Environmental Information. https://doi.org/10.7289/V5QV3JJ5.

36. Sellwood BW, Valdes PJ. Jurassic climates. Proc Geol Assoc. 2008;119(1): 5–17.

37. Stanley SM, Luczaj JA. Earth system history. 4th ed. New York: W. H. Freeman and Company; 2015.

38. Wan M, Zhou W, Tang P, Liu L, Wang J. *Xenoxylon junggarensis* sp. nov., a new gymnospermous fossil wood from the Norian (Triassic) Huangshanjie Formation in northwestern China, and its palaeoclimatic implications. Palaeogeogr Palaeoclimatol Palaeoecol. 2016;441: 679–687.

39. Chinnappa C, Rajanikanth A. Early Cretaceous flora from the Pranhita-Godavari Basin (east coast of India): Taxonomic, taphonomic and palaeoecological considerations. Acta Palaeobot. 2017;57(1): 13–32.

40. Francis JE. The seasonal environment of the Purbeck (Upper Jurassic) fossil forests. Palaeogeogr., Palaeoclimatol., Palaeoecol. 1984;48: 285–307.

41. Morgans HS. Lower and Middle Jurassic woods of the Cleveland basin (north Yorkshire), England. Palaeontology. 1999;42(2): 303–328.

42. Xie A, Gee CT, Bennis MB, Gray D, Sprinkel DA. A more southerly occurrence of *Xenoxylon* in North America: *X. utahense* Xie et Gee sp. nov. from the Upper Jurassic Morrison Formation in Utah, USA, and its paleobiogeographic and paleoclimatic significance. Rev. Palaeobot. Palynol. 2021;291: 104451.

43. Keller AM, Hendrix MS. Paleoclimatologic analysis of a Late Jurassic petrified forest, southeastern Mongolia. PALAIOS. 1997;12: 282–291.

44. Pires EF, Guerra-Sommer M. Growth ring analysis of fossil coniferous woods from Early Cretaceous of Araripe Basin (Brazil). An Acad Bras Cienc. 2011;83(2): 409–423.

45. Pires EF, Guerra-Sommer M, Scherer CM dos S, Santos AR dos, Cardoso E. Early Cretaceous coniferous woods from a paleoerg (Paraná Basin, Brazil). J South Am Earth Sci. 2011;32(1): 96–109.

46. Spicer RA, Parrish JT. Latest Cretaceous woods of the Central North Slope, Alaska. Palaeontology. 1990;33(1): 225–242.

47. Falcon-Lang HJ. Growth interruptions in silicified conifer woods from the Upper Cretaceous Two Medicine Formation, Montana, USA: Implications for palaeoclimate and dinosaur palaeoecology. Palaeogeogr Palaeoclimatol Palaeoecol. 2003;199(3–4): 299–314.

48. Vajda V, Linderson H, Mcloughlin S. Disrupted vegetation as a response to Jurassic volcanism in southern Sweden. Geol Soc Spec Publ. 2016;434(1): 127–147.

49. Decombeix AL, Serbet R, Taylor EL. Under pressure? Epicormic shoots and traumatic growth zones in high-latitude Triassic trees from East Antarctica. Ann Bot. 2018;121(4): 681–689.

50. Franzan BC, Franco TW, Stefani G, Pereira MM, de Almeida FQ, Silva VP. Equine fecal inoculum optimization in in vitro fermentation assays of dehydrated roughage. Rev Bras Zootec. 2018;47: e20180006.

51. Clauss M, Nunn C, Fritz J, Hummel J. Evidence for a tradeoff between retention time and chewing efficiency in large mammalian herbivores. Comp Biochem Physiol Part A Mol Integr Physiol. 2009;154: 376–382.

52. Demment MW, van Soest PJ. A nutritional explanation for body-size patterns of ruminant and nonruminant herbivores. Am Nat. 1985;125(5): 641–672.

53. Christiansen P. On the head size of sauropodomorph dinosaurs: Implications for ecology and physiology. Hist Biol. 1999;13(4): 269–297.

54. Wings O, Sander PM. No gastric mill in sauropod dinosaurs: New evidence from analysis of gastrolith mass and function in ostriches. Proc R Soc B Biol Sci. 2007;274(1610): 635–640.

55. Owen-Smith N, Chafota J. Selective feeding by a megaherbivore, the African elephant (Loxodonta africana). J Mammal. 2012;93(3): 698–705.

56. Pradhan NMB, Wegge P, Moe SR, Shrestha AK. Feeding ecology of two endangered sympatric megaherbivores: Asian elephant *Elephas maximus* and greater one-horned

rhinoceros *Rhinoceros unicornis* in lowland Nepal. Wildlife Biol. 2008;14(1): 147–154.

57. Sukumar R. Ecology of the Asian elephant in southern India: I. Movement and habitat utilization patterns. J Trop Ecol. 1989;5(1): 1–18.

58. Ruggiero RG. Seasonal forage utilization by elephants in central Africa. Afr J Ecol. 1992;30(2): 137–48.

59. Hall-Martin AJ, Erasmus T, Botha BP. Seasonal variation of diet and faeces composition of black rhinoceros *Diceros bicornis* in the Addo Elephant National Park. Koedoe. 1982;25: 63–82.

60. Pellew R. The feeding ecology of a selective browser, the giraffe. J Zool, Lond. 1984;202: 57–81.

61. Tshabalala T, Dube S, Lent PC. Seasonal variation in forages utilized by the African buffalo (*Syncerus caffer*) in the succulent thicket of South Africa. Afr J Ecol. 2010;48(2): 438–45.

62. Larmon JT, Gregory McDonald H, Ambrose S, DeSantis LRG, Lucero LJ. A year in the life of a giant ground sloth during the Last Glacial Maximum in Belize. Sci Adv. 2019;5(2): eaau1200.

63. McNab BK. Food habits and the basal rate of metabolism in birds. Oecologia. 1988;77(3): 343–349.

64. Cooper SM, Palmer T. Observations on the dietary choice of free-ranging juvenile ostriches. Ostrich J African Ornithol. 1994;65(3–4): 251–255.

65. Domínguez-Bello MG, Michelangeli F, Ruiz MC, García A, Rodríguez E. Ecology of the folivorous hoatzin (*Opisthocomus hoazin*) on the Venezuelan plains. Auk. 1994;111(3): 643–651.

66. Downs CT, Wirminghaus JO, Lawes MJ. Anatomical and nutritional adaptations of the Speckled Mousebird (*Colius striatus*). Auk. 2000;117(3): 791–794.

67. Bucher EH, Tamburini D, Abril A, Torres P. Folivory in the white-tipped plantcutter *Phytotoma rutila*: Seasonal variations in diet composition and quality. J Avian Biol. 2003;34(2): 211–216.

68. Fricke HC, Hencecroth J, Hoerner ME. Lowland-upland migration of sauropod dinosaurs during the Late Jurassic epoch. Nature. 2011;480(7378): 513–515.

69. Chin K, Feldmann RM, Tashman JN. Consumption of crustaceans by megaherbivorous dinosaurs: Dietary flexibility and dinosaur life history strategies. Sci Rep. 2017;7(1): 1–11.

70. Woolley L-A, Millspaugh JJ, Woods RJ, van Rensburg SJ, Page BR, Slotow R. Intraspecific strategic responses of African elephants to temporal variation in forage quality. J Wildl Manage. 2009;73(6): 827–835.

Supporting Information

S1 Fig. Gas production of (A) Araucariaceae and (B) Equisetaceae species. Foliage (Araucariaceae) and above-ground shoots (Equistaceae) were collected in the spring of 2004 in Germany (Hummel, unpubl. data).

S2 Fig. Gas production of (A) Araucariaceae and (B) Equisetaceae species. Araucariaceae species were collected in Australia in 2007; Equistaceae species were collected at the Botanical Garden in Zurich, Switzerland in 2011 (Gee, unpubl. data).

S1 Table A. Parameters for the fermentation of Araucariaceae and Equisetaceae species. Foliage (Araucariaceae) and above-ground shoots (Equistaceae) were collected in the spring of 2004 in Germany. For each species, cumulative gas production up to 72 h and fermentative parameters in which $a + b$ is maximum gas production (ml/200 mg DM), and c is rate of gas production are given (Hummel, unpubl. data).

S1 Table B. Parameters for the fermentation of Araucariaceae and Equisetaceae species. Araucariaceae species were collected in Australia in 2007; Equistaceae species were collected at the Botanical Garden in Zurich, Switzerland in 2011. For each species, cumulative gas production up to 72 h and fermentative parameters in which $a + b$ is maximum gas production (ml/200 mg DM), and c is rate of gas production are given (Gee, unpubl. data).

Click here to access/download;Figure;Fig1.tif

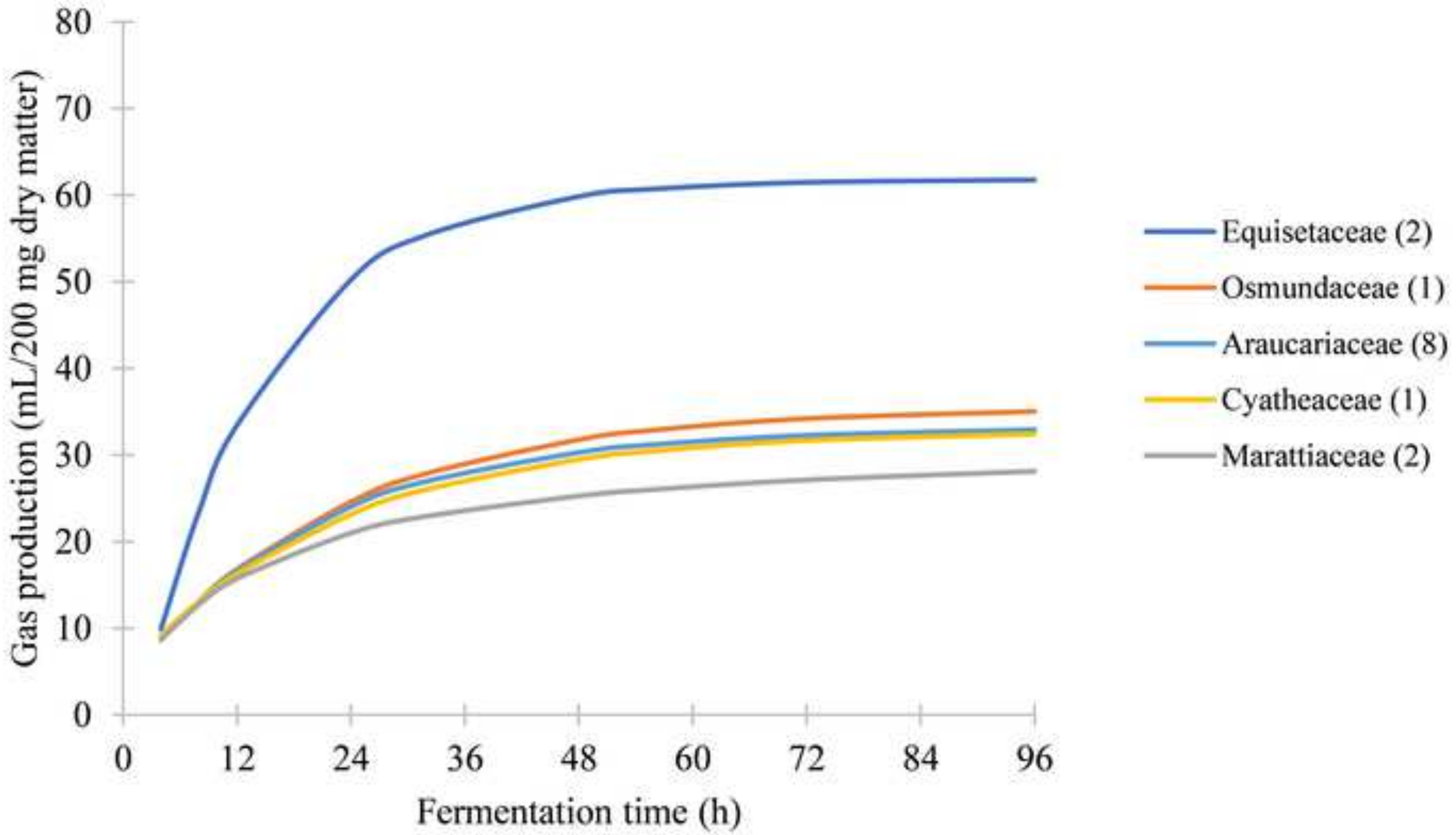

Figure2

Click here to access/download;Figure;Fig2.tif

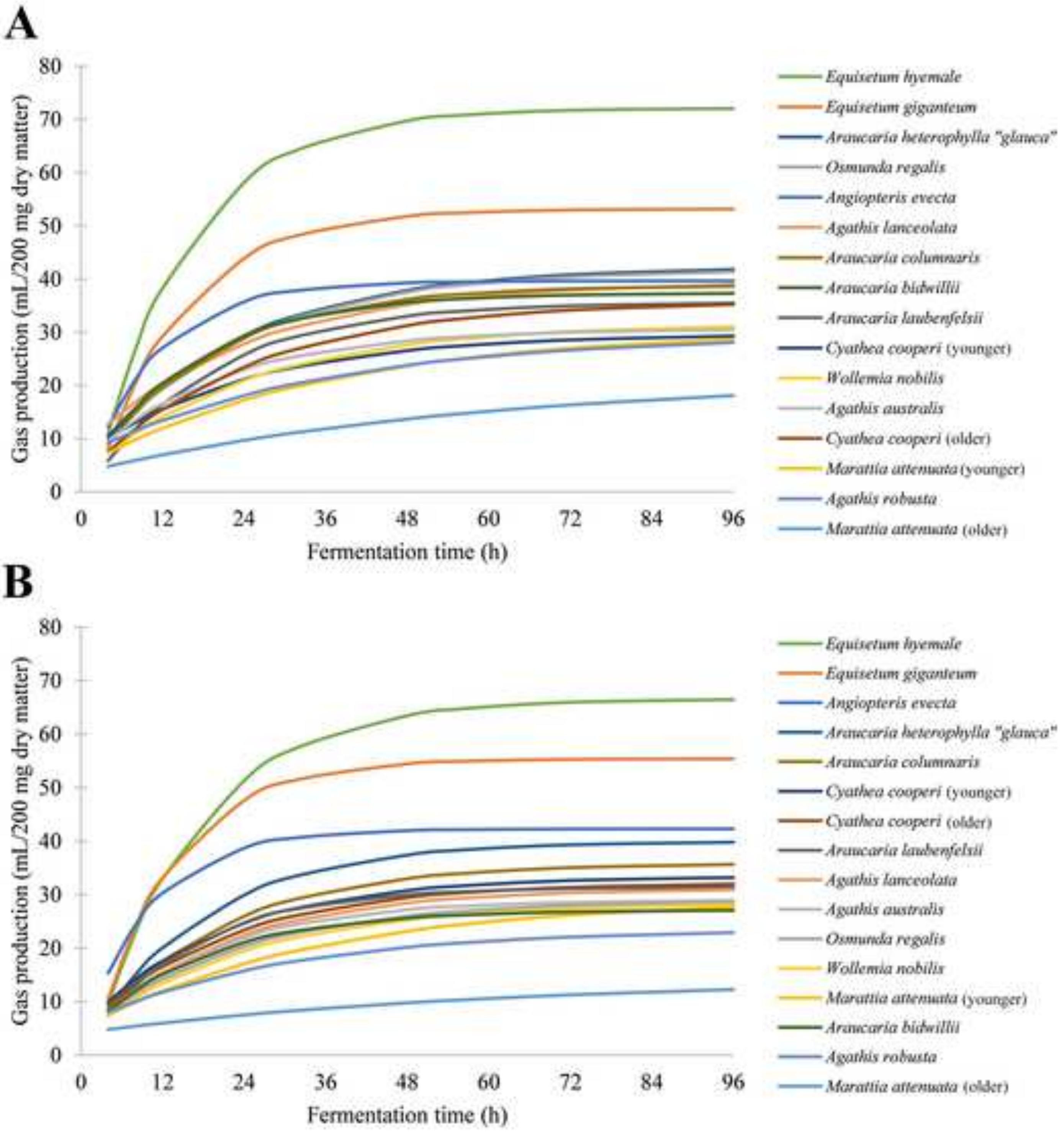

Figure3

Click here to access/download;Figure;Fig3.tif

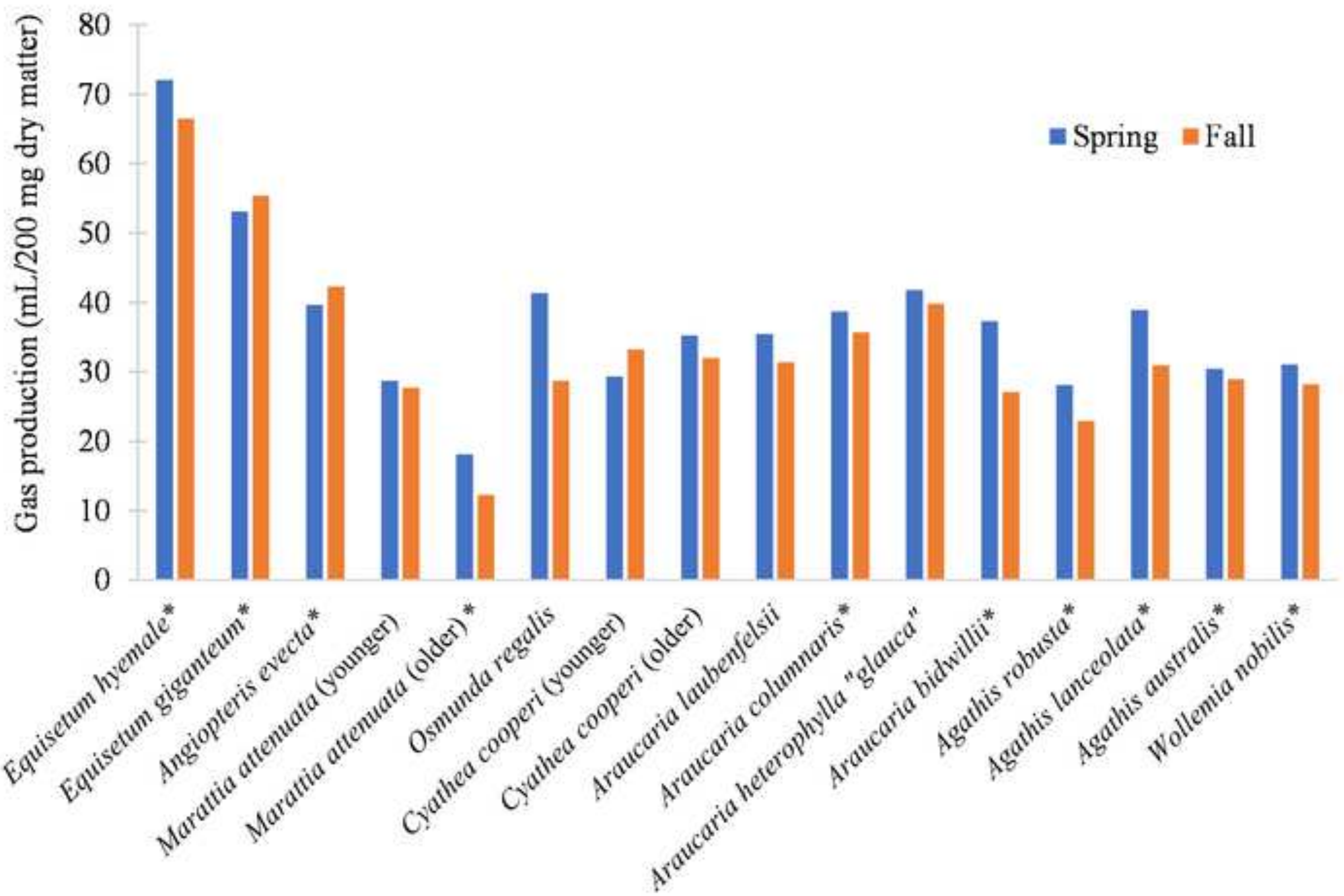

Figure4

Click here to access/download;Figure;Fig4.tif

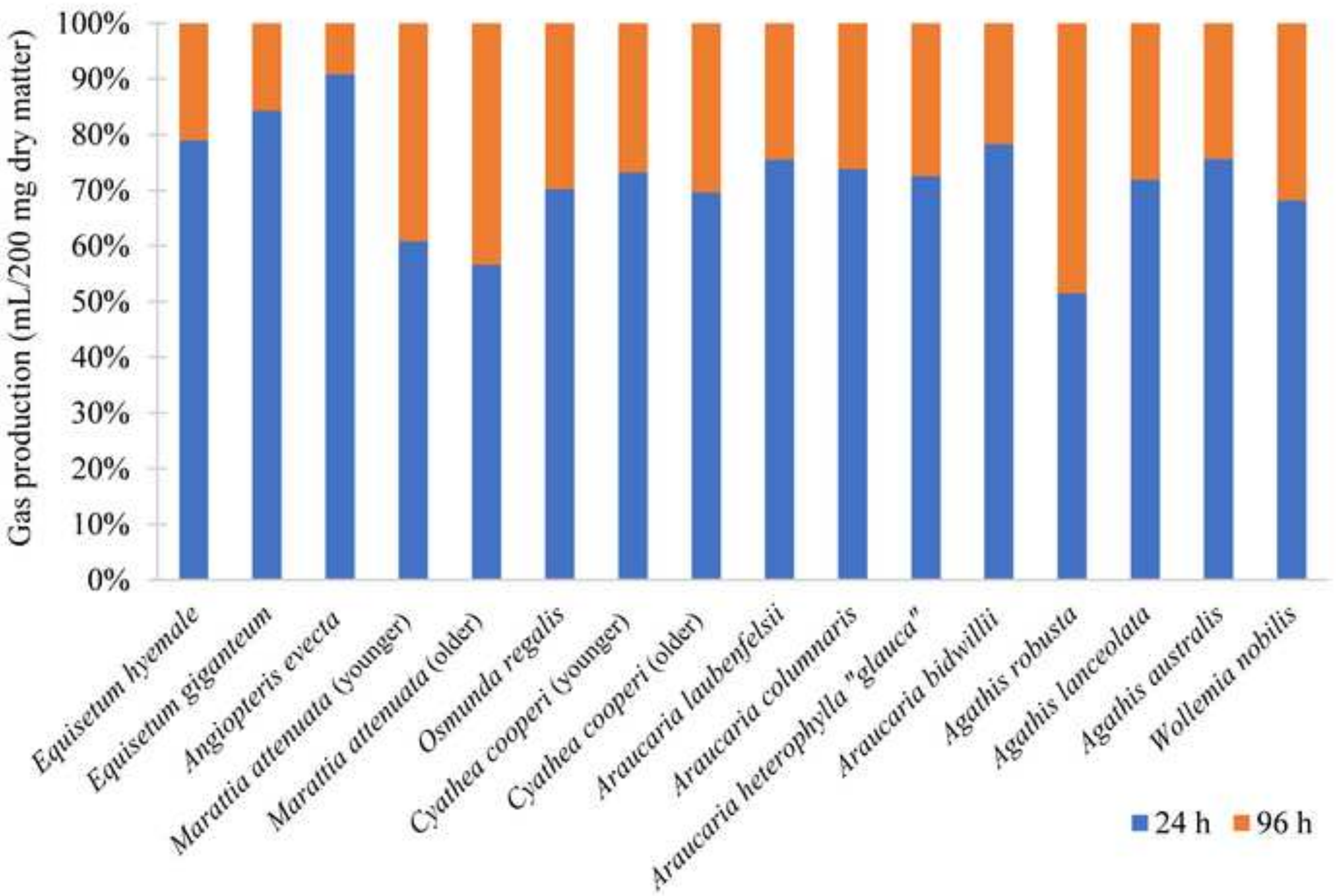

Figure5

Click here to access/download;Figure;Fig5.tif

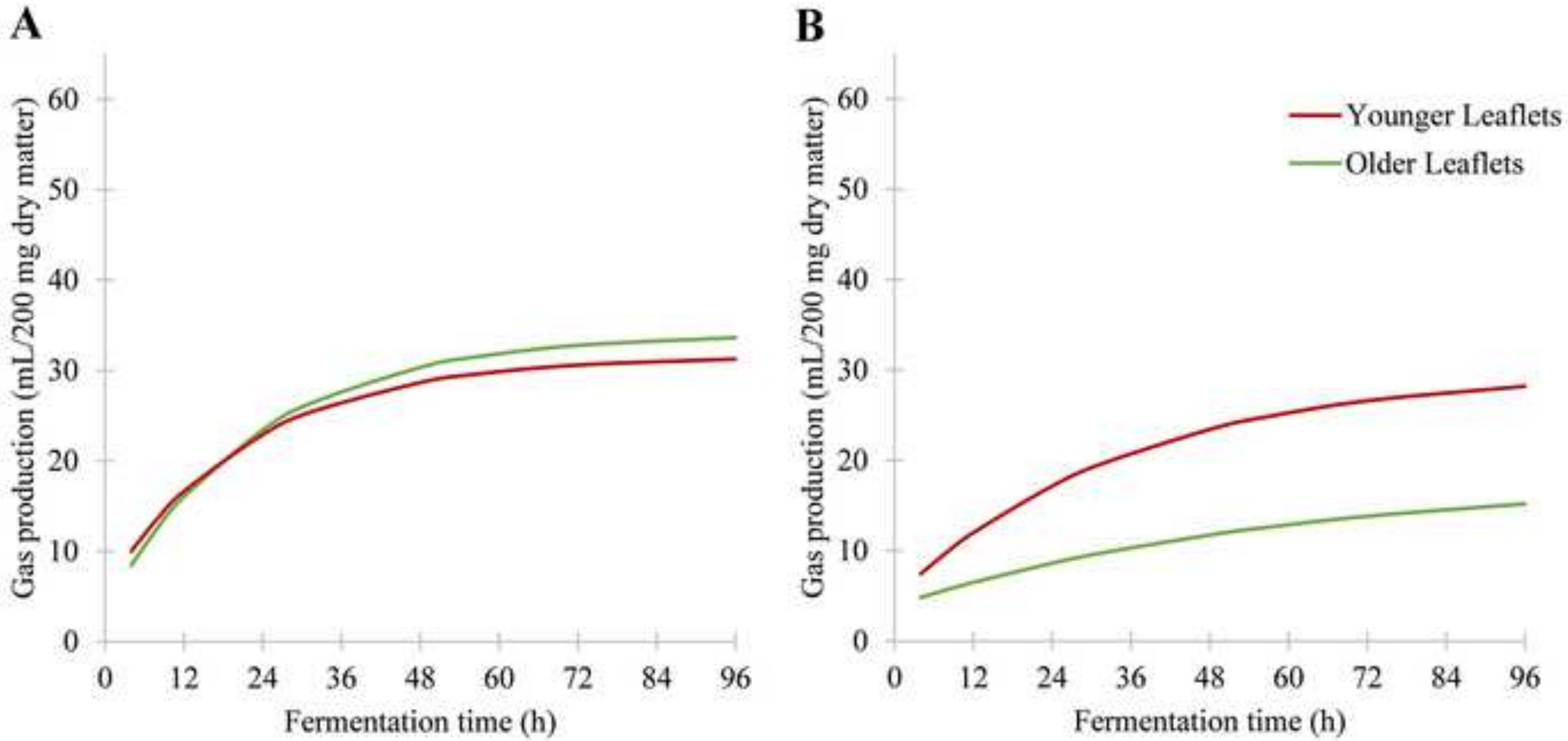

Figure6

Click here to access/download;Figure;Fig6.tif

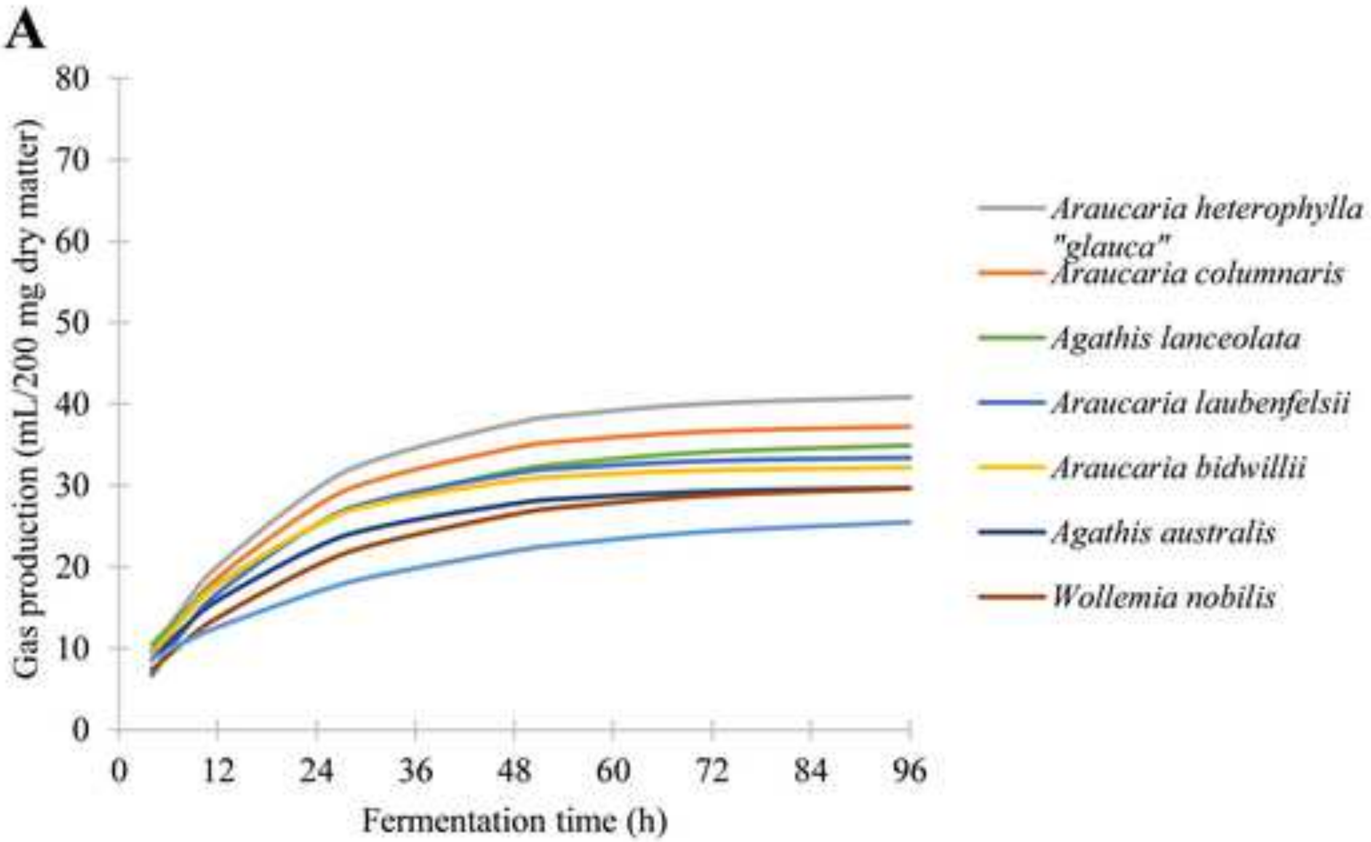

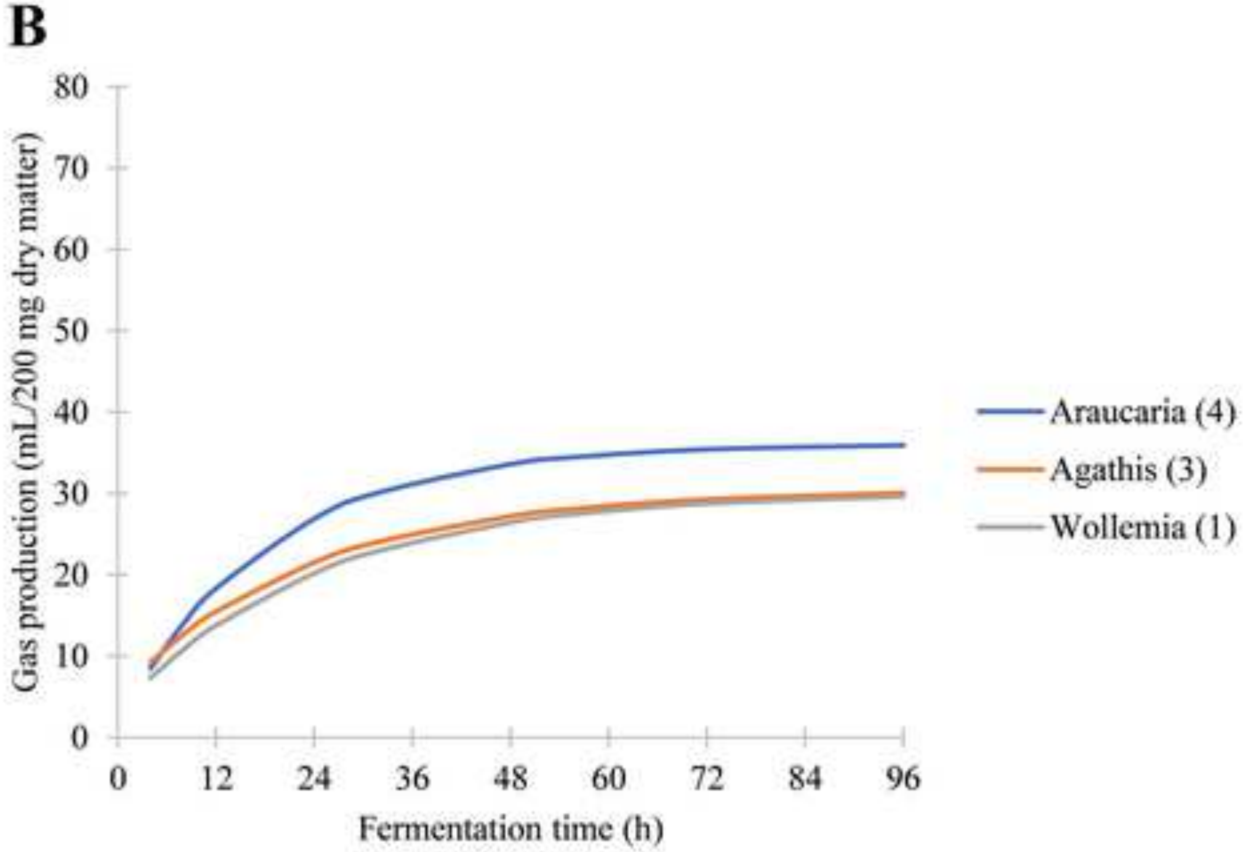

Click here to access/download
Supporting Information
S1 Table A.docx

Click here to access/download
Supporting Information
S1 Fig.tif

Click here to access/download
Supporting Information
S1 Table B.docx

Click here to access/download
Supporting Information
S2 Fig.tif
