# Supplementary material for: Digestibility of dinosaur food plants revisited and expanded: Previous data, new taxa, microbe donors, foliage maturity, and seasonality
Source: PLoS One. 2023 Dec 15;18(12):e0291058. doi: 10.1371/journal.pone.0291058 (PMC10723699; doi:10.1371/journal.pone.0291058)
Supplement: Attachment — Submitted filename: Response to Reviewers.docx [file pone.0291058.s007.docx]

Reply to Reviewers’ Comments on PONE-D-22-20691

Howell, Gee, Böttger, Südekum: Digestibility of dinosaur food plants revisited and expanded: Previous data, new taxa, microbe donors, foliage maturity, and seasonality

We sincerely thank the editor(s) and both reviewers for their review of this manuscript. Following the suggestions of the reviewers, we have added text and references where necessary in order to more clearly explain certain points. All comments have been addressed and the appropriate revisions made.

**Reviewer #1:**

Thank you for the opportunity to review this manuscript. This manuscript is suitable to be published in PloS One.

We are grateful for the positive review of our manuscript and appreciate all of your comments up to this point.

**Reviewer #2:**

The paper: “Digestibility of dinosaur food plants revisited and expanded: Previous data, new taxa, microbe donors, foliage maturity, and seasonality” by Mariah M. Howell et al., has been revised by the authors according to the reviewer suggestions (or explaining their positions). Despite some points remain arguable, I agree with the new version, but still, I have some questions and suggestions for the authors.

We appreciate your thoughtful comments and suggestions during the review of our paper. We agree with nearly all comments that were made, and we have added references or clarifications within the text according to your suggestions.

Introduction:

Lines 71-72, …rumen liquid… and …intestinal microflora… do not get along;

This is a good point. We have replaced “intestinal microflora” with “ruminal microflora” in the sentence.

Lines 75-76, I do not understand the comment, because obviously ash content allow only to know the organic matter content;

You are right that this was confusing. We have added further context to explain the comparison to this study.

“This approach contrasts to the ash test that was used by Weaver [5], which quantified the caloric value of both the digestible and indigestible fractions [1]. With the ash test, it was concluded that cycads were the best source of energy for sauropods, while *Equisetum* was the worst [5].”

Discussion:

Line 292, perhaps it would be better to add “available” before energy;

We agree and have added the word “available” to the sentence:

“…the Araucariaceae consistently yield high quantities of available energy…”

Line 294, do you have an idea about this possibility?

While the digestive properties of ancient taxa cannot be estimated because of the nature of fossilization, we have included more discussion about the likelihood of the conservation of these properties based on morphological similarity:

“Although chemical information of this type is not preserved, the structural morphology of *Equisetum*, for example, has remained largely unchanged since the Middle Triassic [20], suggesting that the digestive properties may also have been conserved through time.”

Line 303-304, the highest…maximum gas production… not correct!

Instead of using the “maximum gas production” value here, we have changed it to refer to the 96-hour gas production value. In this case, *Equisetum hyemale* had the greatest 96-hour gas production of any species in both the spring and fall sets (Tables 2 and 3).

Line 312, …to other the species…?

Thanks for catching this; we’ve now fixed the sentence to read, “Compared to the other species…”

Lines 325-327, the sentence does not appear complete;

This sentence has been rewritten to be clearer and more concise:

“Although the high silica content of *Equisetum* [cf. 21] is thought to inhibit the digestibility of foliage [22], the overall high performance of this genus reiterates that it would have been a consistently nutritious option for herbivorous dinosaurs.”

Lines 429-438, some comments are quite obvious and last sentence is not clear if it is only speculation (useless);

As this paper is of interest to paleontologists that have no background in animal nutrition, we felt it was appropriate to include the information here. Often, there are questions from paleontologists about whether the results of the Hohenheim gas test can vary between individuals or species, so this comparison was included. This is a good point about the last sentence, so we have added a reference and made it more obvious that is a current hypothesis:

“As such, Hummel and Clauss [28] hypothesize that an animal that is accustomed to feeding on the plants analyzed here would most likely have gut flora that is even more efficient at fermenting such material than the gut flora taken from animal feeding on standardized angiosperm foliage.”

Lines 468-473, please to better explain these concepts;

We agree that these ideas needed further explanation and have now added more information to support them. Thanks for pointing this out.

“Although sauropod dinosaurs were not ruminants like the donor species used in this experiment, which have a highly derived digestive system consisting of multiple stomachs to facilitate several oral processing and digestive phases, and it is unlikely that they would have been foregut fermenters [28], the in vitro nature of the Hohenheim gas test minimizes the involvement of the unique digestive anatomy of ruminants while still simulating the fermentative processes that would have occurred within the gut. Because of the rarity of soft tissue evidence in dinosaur fossils, there is no direct evidence as to the nature of sauropod digestive systems; however, Hummel and Clauss [28] suggest that hindgut fermentation is the most likely form of plant digestion in sauropod dinosaurs because a foregut fermenting system is complex to evolve and would not support a high intake diet nor lack of chewing [28]. Here, the Hohenheim gas test uses foregut-fermenting ruminants as the source of digestive microbes, but it has been found that microbial populations and their functions are highly similar in most animal species, which allows for ruminant results to be applied to non-ruminant species [53].”

Lines 491-494, These concepts are extremely simplified, because dry matter intake is affected both by physical and chemical factors; furthermore, I insist that 30 kg of dry matter intake for a 10.8 t animal is “nothing” (an elephant has a DM intake of 1% of its body weight: 40 kg for 4.0 t).

In a mammalian system, these estimates would be unlikely; however, the current consensus is that adult sauropod dinosaurs likely had a metabolic rate of approximately half of a mammalian, tachymetabolic rate (280 kJ ME/kg BW^0.75^ rather than 550 kJ ME/kg BW^0.75^), which accounts for the unexpectedly low amount of dry matter cited here. However, this is a very good point to raise, and we have included the information about metabolic rates so that the estimates can be considered in an appropriate context:

“For adult sauropods, Hummel et al. [1] estimates the amount of consumed dry plant matter sauropods of different body masses and metabolic behaviors would have required based on the energetic density of the plant material, suggesting that a highly digestible diet would allow for 40% less consumed plant mass than a low-quality diet. In their estimates, the number of kilograms of dry matter required varies according to different potential metabolic rates. For example, if they possessed a similar metabolic rate to reptiles (55 kJ ME/kg BW^0.75^), a 70 t sauropod would need as little as 26 kg of high-energy dry matter. However, Hummel et al. [1] believe this to be unrealistic. Therefore, metabolic rates of 280 kJ ME/kg BW^0.75^ (requiring 64 kg DM/day of high energy food or 106 kg DM/day of low energy food) and a mammalian rate of 550 kJ ME/kg BW^0.75^ (requiring 125 kg DM/day of high energy food or 209 kg DM/day of low energy food) were also calculated [1]. Similarly, using an intermediate metabolic rate of 280 kJ ME/kg BW^0.75^, Gill et al. [12] calculates that a 10.8 t Diplodocus would only need to need 23.8 kg of Equisetum hyemale per day, as opposed to 33.2 kg of the fern species Polypodium vulgare.”
